# What are we learning about HIV testing in informal settlements in KwaZulu-Natal, South Africa? Results from a randomized controlled trial

**DOI:** 10.1371/journal.pone.0257033

**Published:** 2022-03-08

**Authors:** Stephanie R. Psaki, Julie Pulerwitz, Brady Zieman, Paul C. Hewett, Mags Beksinska

**Affiliations:** 1 Population Council/Project SOAR, New York, New York, United States of America; 2 Population Council/Project SOAR, Washington, DC, United States of America; 3 Population Council/Project SOAR, Blantyre, Malawi; 4 MatCH Research Unit, Department of Obstetrics and Gynaecology, Faculty of Health Sciences, University of the Witwatersrand, Johannesburg, South Africa; South African Medical Research Council, SOUTH AFRICA

## Abstract

**Background:**

Recent evidence highlighting high HIV incidence and prevalence in informal settlements suggests that they are environments that foster HIV risk. Given growing urbanization in sub-Saharan Africa, there is a critical need to assess the successes and challenges of implementing HIV testing, prevention and treatment interventions in these contexts.

**Methods:**

We randomly selected a household-based sample of 1528 adult men (18–35) and women (18–24) living in 18 randomly selected communities in KZN, South Africa. After the baseline interview, communities were randomized to one of three intervention rollout arms in a stepped wedge design. At approximately 8-month intervals, the *Asibonisane Community Responses* Program (and in particular the implementation of *Stepping Stones*, a participatory HIV prevention program focused on strengthening relationships and communication) was rolled at by intervention phase. Using data from this evaluation, we describe levels and trends in HIV testing and treatment during follow-up, and we use fixed effects models to estimate the effects of participation in the program on testing.

**Results:**

Study respondents reported high levels of economic insecurity and mobility, and men report various HIV risk behaviors including about 50% reporting multiple partnerships. About two-thirds of respondents (73% of women, 63% of men) had been tested for HIV in the last six months. Among those living with HIV, treatment levels were high at baseline, and almost universal by endline in 2019. Program participation led to a 17% increase in the probability of testing for women (p<0.05) but had no effect on testing for men due, in part, to the fact that the program did not reach men who were least likely to be tested, including those who had migrated recently, and those who had never been tested at baseline.

**Conclusions:**

Near universal HIV treatment use demonstrates positive trends in access to some HIV services (including treatment as prevention) in these communities. Stepping Stones had positive effects on HIV testing for women, yet barriers to HIV testing remain, especially for men. Redoubled efforts to reach men with testing are vital for improving HIV outcomes for both men and their partners.

## Introduction

Informal settlements, where communities are formed of ad hoc and improvised housing as residents seek opportunities for employment in the surrounding area, are increasingly a major part of the African landscape, as African cities continue to grow. Most (55%) of sub-Saharan African urban dwellers now live in informal settlements—a proportion that is notably larger than other regions [1]. South Africa is one of the most urbanized countries in sub-Saharan Africa, with a substantial and growing population living in informal settlements [2]. KwaZulu-Natal (KZN) province, a major epicenter of the South African HIV epidemic, where approximately 37% of adult women and 19% of adult men are living with HIV [3, 4], has large informal settlements. According to the most recent census, about 12% of the population of South Africa was living in informal settlements [5]. While many residents are recent migrants with a high degree of mobility, some live their entire lives in these communities [6]. Since the settlements are unplanned, they are often underserved by municipal services, are under-represented politically, and lack accessible healthcare and economic opportunities [7]. This environment can translate into unique challenges and barriers in terms of accessing HIV services and care.

A recent study was conducted to assess HIV prevalence across geographies in South Africa, utilizing four cross-sectional household surveys, from 2002 to2012 [8]. Findings showed a consistent pattern of the highest HIV prevalence within urban informal settlements as compared to urban formal, or rural, settings. Urban informal settlements were also more frequently associated with HIV risk behaviors, such as engaging with two or more sexual partners in the past year. Other nearby countries have also demonstrated higher HIV prevalence in informal settlements. For example, in Namibia’s capital city, Windhoek, hotspot mapping identified informal settlements as having particularly high HIV incidence [9]. These patterns reveal a critical need to assess the successes and challenges of implementing HIV interventions in this context.

The government of South Africa’s National Strategic Plan for HIV, TB and STIs (2017–2022) summarizes the country’s strategy to provide comprehensive HIV prevention and care services nationwide. The strategy describes concentrated efforts to saturate areas with high-impact prevention and treatment services, and to address the social and structural drivers of HIV infection. On the service side, this includes enhancing utilization of HIV testing services, and linkages to care and treatment for those who test positive [10]. In addition, HIV prevention has rapidly evolved in recent years with development of high-impact interventions such as treatment as prevention (TasP) [11], treating PLHIV with ARV in order to decrease transmission events, and HIV preexposure prophylaxis (PrEP) [12]. Transmission events involve both those newly acquiring HIV as well as the large numbers who are unaware of their status and are not virally suppressed [13]. Testing is thus the key entry point into the care continuum for both those living with HIV as well as to prevent acquiring HIV [14].

Community-based HIV programs are seen as promising approaches to reaching communities in informal settlements and promoting HIV testing, prevention, and treatment. These programs often have multiple components, including group discussion, where groups of men and women engage in learner-centered and interactive activities that foster critical reflection about key topics related to HIV. These topics can range from basic prevention information to contextual issues that place individuals at risk, such as relationship dynamics, gender norms and structural inequalities [15, 16]. Stepping Stones is one such example. Developed for use in Uganda (in 1995), the program has been applied in over 40 countries, with hundreds of thousands of individuals, and adapted for dozens of settings. Stepping Stones is a participatory group intervention that aims to prevent HIV and violence by building stronger, more gender equitable relationships, and promoting communication skills. A rigorous evaluation conducted in the rural Eastern Cape province of South Africa, and using a randomized controlled trial (RCT) methodology, found a reduction in sexually transmitted infections (i.e., HSV-2), men’s reported perpetration of intimate partner violence, transactional sex, and alcohol abuse, although it failed to find a reduction in HIV incidence [17]. A systematic review published in 2013, where the authors extracted from several databases and drew upon the ‘grey’ literature, found evaluations that demonstrated increases in condom use and the communication of HIV information to partners, and reductions in multiple partners, although these results were inconsistent [18]. Overall, strengthened partner communication was the most consistent finding. An adaptation of the Stepping Stones program—which added a component to strengthen livelihoods (i.e., Creating Futures)—was recently evaluated via an RCT in KwaZulu-Natal, South Africa. The study found that women reported increased earnings and men reported reduced IPV perpetration, although women did not report a reduction in IPV experience [19].

Less is known about the effects of Stepping Stones on use of HIV services including HIV testing. Only one published study to our knowledge explored program effects on HIV testing. A mixed-methods evaluation in Karnataka, India, with 20 intervention and 20 control villages, found both an increased likelihood to consider HIV testing as well as actual increased HIV testing (p < 0.01) [20]. Conceptually, facilitating relationship and communication skills—as Stepping Stones does—will improve participants’ ability to talk about sexual matters and HIV with their partners, including the need for HIV testing. And, facilitating gender equity and shared decision-making power—as Stepping Stones also does—will lead to more successful negotiation of desired outcomes, including HIV testing.

### Objectives of this study

Given the increasing number of people living in informal settlements in Africa, and the high HIV prevalence rates found in these settlements, there is a critical need to assess the successes and challenges of implementing HIV testing, prevention, and treatment interventions in these contexts. This study contributes to existing knowledge by reporting on HIV-related service utilization for men and women over four rounds of data collection, beginning in 2017, as well as the effects of a community-based HIV prevention intervention centered around strengthening relationships and communication (Stepping Stones) on HIV testing. We also explore the characteristics of who is being tested, and who programs are missing. Building the global evidence base on these questions is vital for reaching communities with services, and for improving HIV outcomes for men and their partners.

## Materials and methods

### Intervention

The Asibonisane Community Responses (CR) program was designed to promote uptake of HIV services and transform gender norms to create an enabling environment for HIV prevention behaviors among adult men and women. It was supported by PEPFAR/USAID and implemented by the Centre for Communication Impact (CCI) in partnership with Dramaide and The Valley Trust (TVT). The target population for the five-year CR program (2014–2019) was young men (ages 15–35) and women (ages 15–24) living in informal settlements. The CR program included several components: a ten-session version of Stepping Stones, single-session educational meetings for community members that addressed a range of HIV prevention topics, and other periodic community-based meetings tailored for men that addressed sexual and gender-based violence (SGBV).

The core component of the CR program was a ten-hour version of Stepping Stones, a widely used life skills training intervention focused on curbing gender-based violence (GBV) and reducing HIV risk [21]. The manual-based curriculum addresses issues such as communication about HIV, relationship skills, and assertiveness, and encourages participants to engage in critical reflection through role-playing and group dialogue. Previous evaluations of Stepping Stones in South Africa found significant reductions in intimate partner violence and herpes simplex virus, and improvements in couple communication and negotiation, among other positive outcomes [17, 22].

The single session meetings were two to three hours in length, in which between 20 and 40 men and women participated, by age group. During these meetings, facilitators shared a range of HIV information, including on prevention and transmission. SGBV prevention meetings included community dialogues and workshops for men, where participants were encouraged to reflect on their own experiences, attitudes and values regarding gender-based or intimate partner violence, HIV/AIDS and human rights. Community members attending the single session meetings were then invited to join the more intensive Stepping Stones groups.

### Study design and sample selection

This study was designed as a stepped-wedge cluster randomized trial with a prospective cohort sample to evaluate a community-based HIV prevention program (*Asibonisane* Community Responses) being implemented in informal settlements in the eThekwini and Ugu sub-districts of KZN. In a stepped-wedge evaluation design, clusters (communities) are assigned to cross over from control to intervention at different time points [23]. The stepped-wedge evaluation was designed to be implemented in a subset of communities within the broader CR program implementation so that the evaluation would not impede the general rollout of the CR program in non-evaluation areas. However, as expanded upon later, due to issues with the program rollout (as planned in the study protocol), we analyze the results as a quasi-experimental study rather than an RCT.

Based on a study design with three implementation steps and four rounds of data collection, a power criterion of 0.80, alpha coefficient of 0.05, intra-cluster correlation of 0.05, and a conservative assumption of baseline prevalence (45%) of key outcomes, the minimal sample size required at the end of the study was approximately 1,260 individuals (630 men, 630 women), distributed across 18 clusters with 35 men and women in each clusters. Assuming 15% loss to follow up, an estimated baseline sample size of 1,500 individuals (750 men, 750 women) was specified. A minimum 10% percentage point effect size (change due to intervention) was specified for the primary outcome indicators. Sample size estimates were stratified by sex, such that the study was powered to draw inferences about impact separately for males and females, who were sampled from different households.

USAID and CCI identified priority wards for the CR program within eThekwini and Ugu sub-districts in KwaZulu-Natal province based on the prevalence of HIV in the area and the need for HIV prevention programming. Wards are geopolitical subdivisions of municipalities. Although defined largely for electoral purposes, wards are a geographic level of demarcation use by the South Africa National Census. Within the selected wards, informal settlements were identified for targeting by the CR program. Depending on size, the informal settlements were further sub-divided into smaller clusters for the impact evaluation. A minimum number of households and clear demarcations (roads, highways, paths) were used as requirements for the area to be identified as a sampling cluster for the evaluation and subsequently stratified by high and low-density areas. From the full list of approximately 80 informal settlements, 18 were randomly selected proportional to size using Stata version 14.2 by PH and BZ. Baseline data collection began in early 2017 in all sites. After each of three ten-month program rollout periods, follow-up surveys (rounds 2 through 4) were conducted among all study participants. In rounds 1 and 2 of data collection all interviews were conducted in person. Due to high loss to follow-up between those rounds, we amended our protocol to conduct telephone interviews when participants were unable to be interviewed in person.

**Fig 1:**
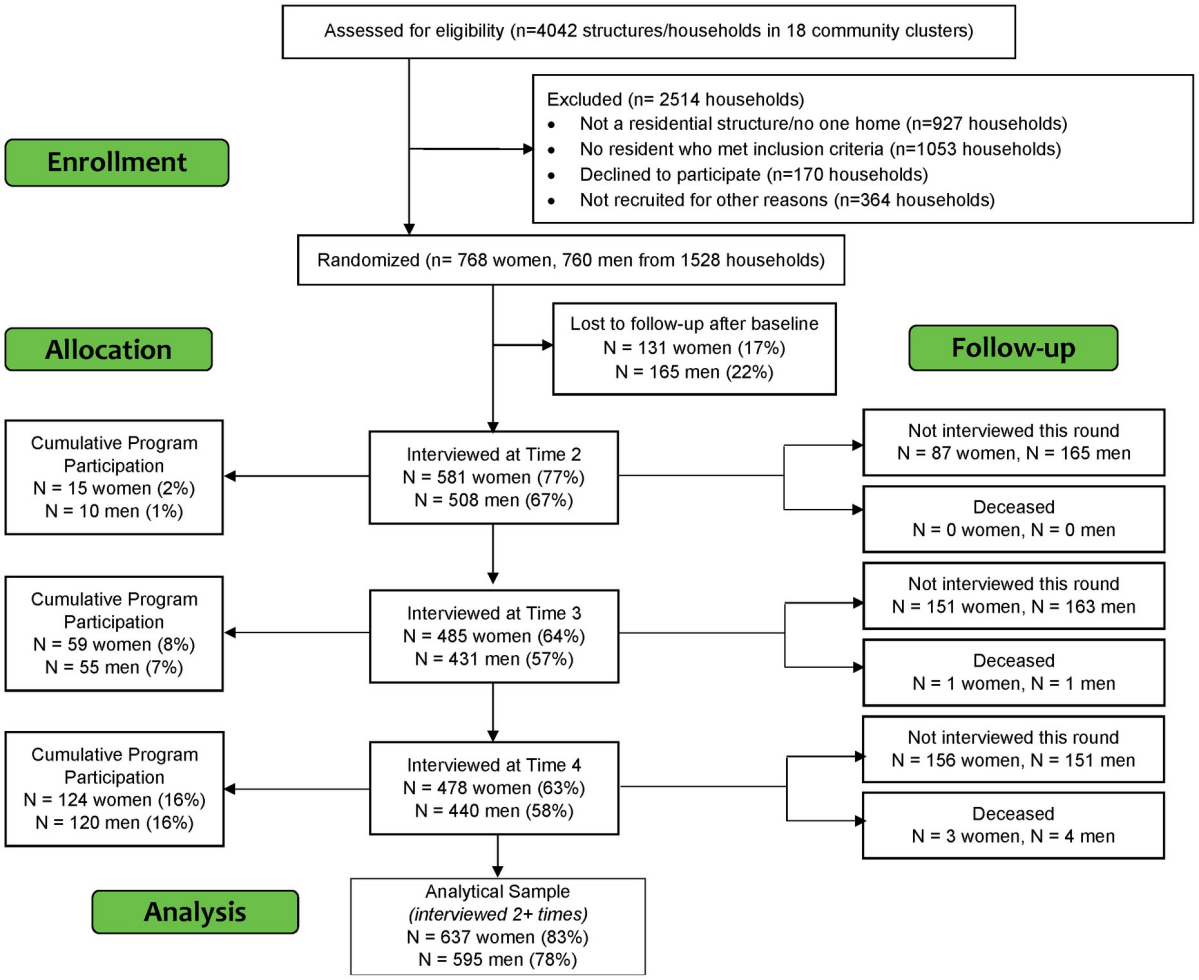
CONSORT Flow Diagram.

Eligible study participants included men (aged 15–35), and young women (aged 15–24). The evaluation was limited to men aged 18–35 and women aged 18–24 as the highest HIV incidence rates are found in these age groups. Additional criteria for eligibility were: living in the study community, able to read English or the local language, willing and able to give informed consent, willing to participate in three additional interviews at ten month intervals, willing to provide study team with contact information for follow-up interviews, and did not reasonably foresee moving out of the area during the study period. Eligible men and women were sampled from households within the informal settlements through a process of mapping, random household selection, resident enumeration, and participant selection. Within the 18 selected clusters, 4042 structures (out of a total pool of 20454 structures) were randomly assessed for eligibility based on an algorithm used by the data collection tablet. Of those, an additional 2514 structures were excluded (see Fig 1, Enrollment). From the remaining 1528 households, 768 women and 760 agreed to participate in the study. Each of the 18 clusters was then randomly assigned to program rollout group using Stata version 14.2 by BZ, and community leaders were informed of their assignment by the MatCH Research Unit study team, in coordination with the local organizations implementing the intervention.

### Ethical considerations

Before administering the baseline questionnaire, the interviewer explained the study and its objectives to the selected participant. If the participant was still interested, the interviewer either read aloud or asked the participant to read the appropriate informed consent document. The consent form described the risks and benefits associated with study participation. Participants were informed that they could refuse to answer any questions, stop the interview at any time, or withdraw participation in the study. Once all questions were answered to the participant’s satisfaction, the interviewer asked the participant to provide written consent. To complete the informed consent process, the interviewer signed the consent form and provided a copy to the participant. Informed consent forms and questionnaires were available in English and Zulu, and interviewers were available to translate into Xhosa as needed. One copy of the signed consent form was given to the participant, another original copy was kept in a locked file in MatCH Research Unit’s office, separate from other source documents that would link study data with participant identification.

After obtaining informed consent, the survey was administered in a private setting within the participant’s home or an alternative private location. The interview lasted approximately one hour and was conducted using a tablet, by an interviewer of the same sex as the participant. The most sensitive questions on gender-based violence and HIV status were self-administered. Participants were compensated for their time and for any travel costs based on current participant compensation rates used by MatCH Research Unit in similar studies. Those who enrolled in the cohort study and completed the survey were paid R100 (~$7.00) for each round of data collection.

We worked closely with community members before, during and after data collection to ensure community support. Prior to the start of the study, we engaged ward councilors (local elected officials) in each settlement, each of whom provided a letter of permission before data collection began. We presented the study in advance to the community members through community meetings. Both the ward councilor and staff from the community-based organizations implementing the intervention provided feedback on the study design and content of the questionnaires. They were also involved in the implementation of the study by attending community meetings, assisting with security issues during data collection, explaining the study and intervention to community members, and helping to trace those who moved. After data collection was complete, we disseminated the results through several key stakeholder meetings at the local, provincial, and national levels. The study’s community advisory board planned to continue dissemination once the results were finalized, but this has not been possible due to COVID-19 restrictions. We plan to conduct additional dissemination activities when it is safe to return to study communities.

Prior to the commencement of data collection, the study protocol was published through the ISRCTN Registry (ISRCTN18195524), where it can be accessed online. The protocol was also reviewed and approved by the Population Council’s Institutional Review Board, and the Human Research Ethics Committee of the University of the Witwatersrand. All study participants provided informed consent.

#### Analytical approach

The full study protocol pre-specified a series of key outcomes focused on increased use of HIV prevention and treatment services, reduction in sexual risk behaviors, reduction in the experience and perpetration of SGBV, and decreased support for harmful gender norms. This paper reports on a subset of those outcomes focused on use of HIV prevention and treatment services and sexual risk behaviors. The results on the other outcomes are included in a separate manuscript that is in development.

This paper presents results in two sections. First, we describe the experiences of men and women living in informal settlements in KZN, including demographic characteristics and measures of economic insecurity. We also describe baseline levels of HIV prevalence, testing, and treatment. Second, we explore trends over time in these three outcomes and the extent to which improvements can be attributed to participation in the CR program. We also explore whether the program was able to reach those most in need of services.

Although clusters were randomized to receive the CR program at a certain time, we observed considerable spillover in program participation between study arms. Given departures from the randomized study design, we estimate associations between participation in the CR program and key outcomes using linear random effects and fixed effects models, run separately for men and women.

We estimate trends over time in our outcomes of interest, as well as the relationship between participation in the CR program and HIV testing using the following model:

$${Test}_{t}=\beta_{0}+\beta_{1}P_{t}+\beta_{2}G+\beta_{3}M_{t}{\;+\;\beta }_{4}R{+\;\beta }_{5}D+u+v_{t}$$

Where *Test*_*t*_ represents whether the respondent had been tested for HIV in the last six months at time t (or other key outcomes), and takes a value of 0 or 1; P_*t*_ is participation in the intervention by time *t* (0 or 1); G is rollout group assignment (1, 2, or 3); M_*t*_ is time since last interview (in months); R is a baseline measure of risk, as appropriate (e.g., HIV testing in the last six months at baseline); D is a set of socio-demographic covariates measured at baseline, including level of education and employment status; u represents unobserved determinants of both the outcome and key independent variables (e.g., risk aversion); and v_*t*_ is a random disturbance term. Time (t) represents rounds of data collection, where t = 0 is baseline, and possible values are 0 through 3. We run separate models for men and women.

It is important to recognize the statistical estimation problems that result from selection into the program based on factors that are unmeasured or unobservable. The central problem is that these factors (represented as *u* in the model) may affect both participation in the program and outcomes and behaviors of interest, leading to biased estimates of program impact if selection is not considered. To partially address these limitations, we run both random effects and fixed effects models, the latter of which control for unobserved constant influences from individual, family, and community characteristics [24].

Respondents were asked whether they had ever been tested for HIV, and if so, when was their most recent HIV test, and whether they received the results of that test. Later in the survey, respondents were asked about the results of their most recent HIV test. If they reported that they had tested positive, they were then asked whether they had, *“received medication to treat your HIV called ART*, *or antiretrovirals*, *in the last 6 months”*, and whether they were currently taking any medication received. The HIV testing models exclude those who reported that they were HIV-positive in the previous round. The antiretroviral treatment models are limited to those who reported that they were HIV-positive in the current round. As a result, the samples for models vary, and in some cases we are unable to assess program effects when the sample sizes are too small (for HIV treatment).

Respondents were asked whether they currently had a *“main or ‘straight’ partner”*. If they said yes, they were then asked a series of questions about that partner, and the nature of the respondent’s sexual relationship with that partner, including condom use. After a series of questions about the “main” partner, respondents were then asked, *“Do you have a ‘second partner*?*’ By second partner*, *I mean another partner with whom you have an ongoing relationship*.*”* If they said yes, they were asked the same questions about the second partner. Later in the survey, respondents were also asked how many partners, in total, they had had sex with in the last six months, by partner type (spouse, cohabiting, girlfriend/boyfriend, casual, etc.). If they responded that they had had sex with a casual partner in the last six months, respondents were then asked whether they used a condom the last time they had sex with a casual partner.

We used SurveyCTO to assist with data collection, and all analyses were done in Stata version 14.2.

## Results

### Characteristics of men and women living in informal settlements

At baseline in 2017, we interviewed 760 men and 768 women. Table 1 shows characteristics of the full study sample at baseline, which was representative of men (ages 18–35) and women (ages 18–24) in the study communities. The baseline sample of men and women provides valuable insight into the experiences of adults living in informal settlements in urban KZN. Study participants are highly mobile: 18% of both men and women had lived in their community for less than a year, and 18% of men and 12% of women had slept away from their home for more than 30 days in the previous six months (p<0.01). Compared to women, men were more likely to have lived in their community for more than two years (73% of men vs. 60% of women, p<0.001).

**Table 1:** Baseline characteristics of study population by sex (n = 1528).

	Men	Women	
	(n = 760)	(n = 768)	
*Mean age*	25.1	21.3	***
*Length of time living in community*			***
0–12 months	17.9%	18.3%	
13–24 months	9.4%	21.8%	
25+ months	72.7%	60.0%	
*Slept away from home > 30 days (past 6 months)*	17.5%	12.3%	**
*House construction materials*			***
RDP housing	4.9%	0.9%	
Other brick structure	24.5%	23.2%	
Shack: corrugated iron or wood walls	42.0%	53.4%	
Shack: cardboard or mud walls	25.8%	16.1%	
Other	2.9%	6.4%	
*Completed secondary school or more*	44.5%	48.7%	~
*Partnership type*			***
No partner	40.7%	27.1%	
Live together	9.9%	18.6%	
Live separately	49.4%	54.2%	
*Number of children living at home*			***
None	81.1%	50.4%	
1	8.4%	32.2%	
2	5.1%	10.9%	
3+	5.4%	6.5%	
*Primary source of income (past 3 months)*			***
Unemployed, no income	51.1%	52.6%	
Employed	48.2%	12.4%	
Social grants/Family	0.8%	35.0%	
*Average monthly income (past 3 months)*			***
No income	51.1%	52.6%	
1–500 ZAR	2.0%	27.1%	
501–1500 ZAR	7.8%	13.7%	
1501+ ZAR	28.2%	6.6%	
Refused	11.1%	0.0%	
*Household member did not eat for a day*, *past 4 weeks*			***
Never	67.1%	80.6%	
Once or twice	25.3%	12.9%	
Three times or more	7.6%	6.5%	
*Ownership of assets*			
Radio	60.3%	46.2%	***
Television	55.5%	61.5%	*
Computer	8.7%	7.0%	
Refrigerator	43.9%	51.0%	**
Cellphone	98.9%	98.4%	

Notes: Tests indicate significant differences at baseline between males and females, based on chi-squared tests.

***p<0.001;

**p<0.01;

*p<0.05,

^~^p<0.10

Men and women living in informal settlements also experience high levels of economic insecurity. Overall, more than half of men and women reported earning no income in the past three months. However, men were more likely to be employed (48% of men vs. 12% of women, p<0.0001), and they had higher incomes on average (28% of men vs. 6% of women reported monthly incomes over 1500 ZAR, about 100 USD, p<0.0001). Women were more likely to have children at home (19% of men vs. 50% of women, p<0.0001), and for a third of women their primary source of income was social grants. About 20% of women and 33% of men (p<0.0001) said they or a member of their household did not eat for at least one day in the past four weeks. About two-thirds of men and women reported that they live in a shack, rather than a more permanent structure.

Table 2 includes information about study participants’ reported sexual behavior, including condom use, use of HIV services, and HIV status at baseline. The majority of respondents reported having sex with a primary partner in the previous six months (60% of men, 72% of women, p<0.0001). Slightly more than half of men (54%) and women (55%) reported using condoms at last sex with a primary partner (p>0.05). About half of men reported having sex with two or more partners in the previous six months, and about 60% of men reported using a condom at last sex with a casual partner. In comparison, 7% of women reported having sex with two or more partners in the previous six months, and 52% of women reported using a condom at last sex with a casual partner, although the sample for that estimate was small.

**Table 2:** Baseline behavioral and service use characteristics of study population, by sex (n = 1528).

	Men	Women	
	(n = 760)	(n = 768)	
*Sex with primary partner*, *last 6 months*	60.1%	71.7%	***
*Condom use at last sex with primary partner*	54.1%	55.4%	
*2+ sexual partners*, *last 6 months*	50.5%	7.0%	***
*Condom use at last sex with casual/one-off partner*	59.8%	51.7%	*
*Ever tested for HIV*, *last 6 months*	67.0%	76.9%	***
*Ever tested for HIV*, *lifetime*	90.0%	91.6%	
*HIV-positive (self-reported)*	7.6%	14.3%	*
*Current ART use (among HIV positive)*	95.1%	94.6%	

Notes: Tests indicate significant differences at baseline between males and females, based on chi-squared tests.

***p<0.001;

**p<0.01;

*p<0.05,

^~^p<0.10

Ninety percent of both men and women reported ever being tested for HIV at baseline, with a substantially smaller proportion (although still a majority) reporting a test for HIV in the last six months. A higher proportion of women (77%) than men (67%) had been tested for HIV in the last six months at baseline (p<0.0001). Women in South Africa often get tested during antenatal care, as reflected in the higher level of testing (91%) among women who said they were currently pregnant (not shown). As is consistent with national levels, self-reported HIV prevalence was significantly higher for women than men at baseline (for this sample, 14.3% for women and 7.6% for men, p<0.05). Among those who reported they were HIV-positive, levels of self-reported ART use were high at baseline (95% for HIV-positive men and women).

### Trends and program effects

#### Loss to follow-up

Fig 1 shows how the study sample evolved during follow-up. At baseline we interviewed 760 men and 768 women. We sought to re-interview all women and men who participated at baseline for each of the additional three rounds of data collection. Some respondents were unavailable for one or two rounds but were later interviewed again. The analytical sample includes those who were interviewed at least twice during follow-up, i.e., those who were not lost to follow-up. Cumulative program participation represents those who had ever participated in Stepping Stones or another SGBV activity (defined as participation in our analyses), but does not include those who participated in other Community Responses activities only (such as brief community meetings). By the final round, 22% of male participants and 17% of female participants had been lost to follow-up, defined as never completing another interview after baseline. An additional 4 men (<1%) and 3 women (<1%) were reported deceased. Baseline data collection began in February of 2017 and continued through May 2017; four rounds of data collection were conducted in accordance with the study protocol. We aimed to interview study participants at approximately eight month intervals, but follow-up time varied by individual, ranging from 7.7 months on average between rounds 3 and 4 to 9.2 months on average between rounds 1 and 2, reflecting increased efficiencies at following up the sample over time. As noted, models control for time since last interview to address this variation. The analytical samples for our models assessing trends over time and program effects include those who were interviewed at least twice during follow-up, which is 78% of the baseline male sample (n = 595) and 83% of the baseline female sample (n = 637).

For both men and women, compared to the full baseline sample, those lost to follow-up had lived in the community for less time (0–12 months vs. 13+ months), were slightly less educated, less likely to have a main partner, more likely to be employed, and less likely to own a set of assets (radio, TV, computer, fridge, cell phone). For women, there were no differences in baseline measures of study outcomes. For men, those lost to follow-up were less likely to have reported being HIV-positive at baseline than those in the analytical sample (results not shown).

#### Participation in the CR program

For the purposes of the evaluation, we define program participation in three groups: no participation, low-touch participation (reporting attendance at a single session education meeting or SGBV activities, but not Stepping Stones), and high-touch participation (reporting Stepping Stones participation, with or without other activities). Fig 1 shows the timing of participation by round of follow-up, focused on high touch participation. Although participation should have been evenly distributed across rounds based on the study design, few study respondents participated in the program between rounds 1 and 2 (n = 15 women, 10 men); participation increased in subsequent rounds. We focus our analyses on the high-touch participation group, which included 16% of men and 16% of women by endline. An additional 9% of men and 6% of women were in the low-touch participation group, which we control for in our models. We define program participation this way because by focusing on more intensive intervention components, we are estimating the effect of the intended participation in the program. High-touch participants and non-participants (including both no participation and low-touch participation) were largely comparable, with a few exceptions. Compared to women who did not participate in the program, women who participated were similar on many important characteristics, including measures of socio-economic status (education, household materials, assets), most sexual behaviors (condom use, sex with a primary or second partners), as well as HIV status, testing and treatment. However, compared to non-participants, women who participated in the program had lived in their communities for longer, were more likely to have children living at home, and were less likely to have had sex with 2+ partners in the last 6 months at baseline. Male participants and non-participants were also similar in many ways, including socio-economic status, mobility, types of partners, condom use, and HIV status, testing and treatment. Compared to men who did not participate in the program, men who participated lived in earlier rollout communities (results not shown).

#### HIV testing and treatment

**Fig 2:**
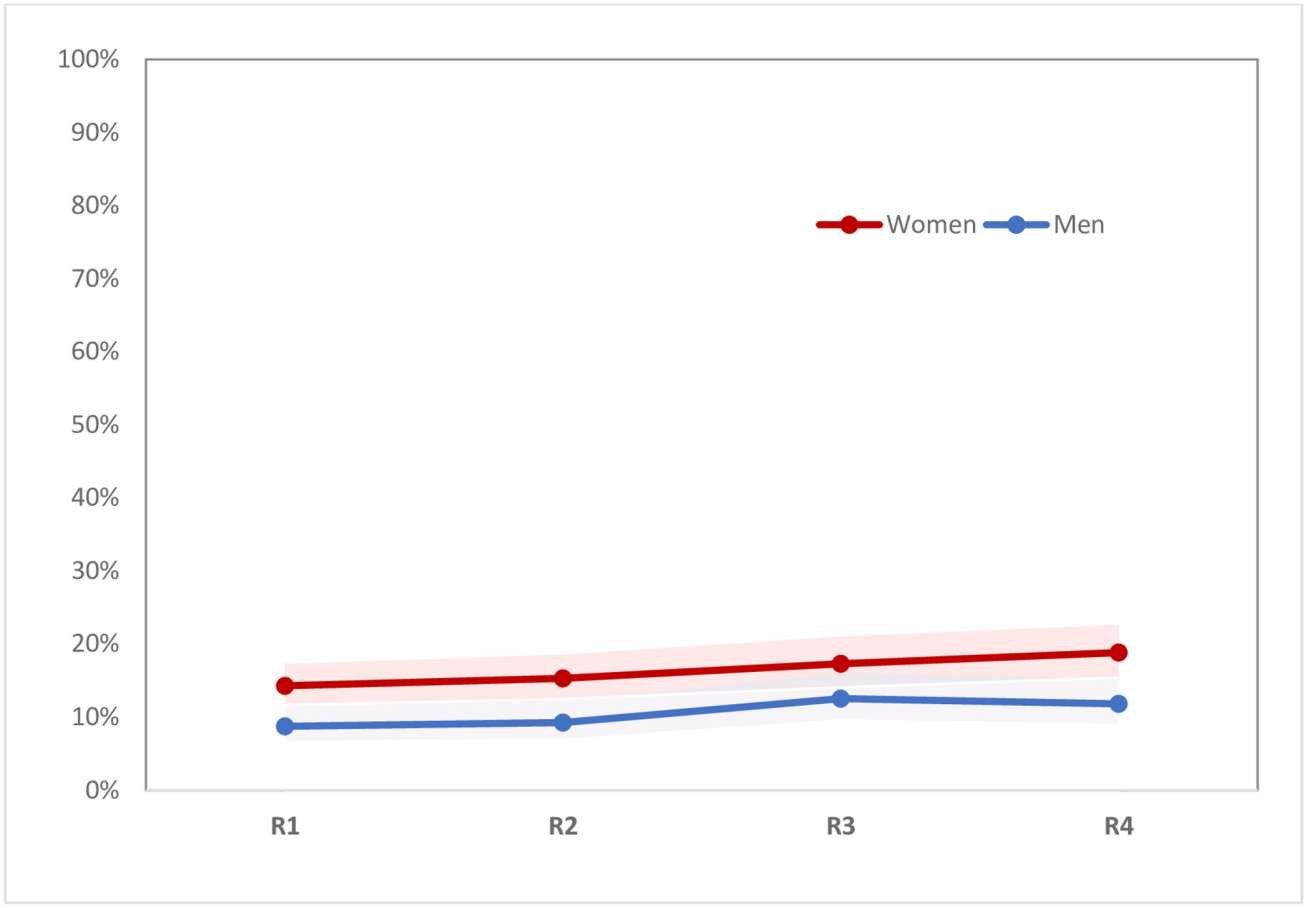
Percent reporting they were HIV-positive, by sex (n = 595 men, 637 women). Note: Figures showing trends include only men and women in the analytical sample, so values may differ slightly from those shown in Table 1.

**Fig 3:**
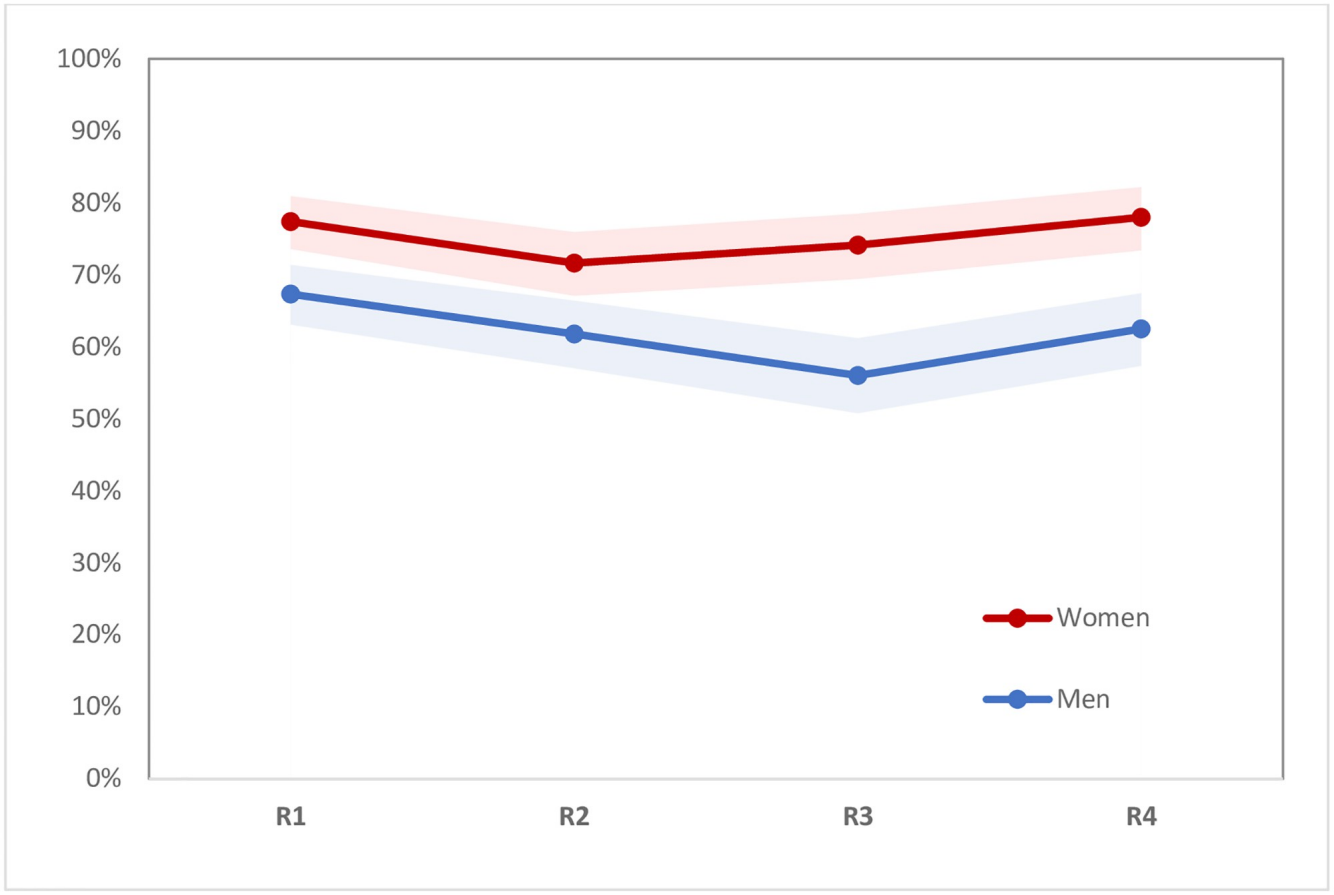
Percent reporting that they were tested for HIV in the last 6 months, and 95% confidence intervals, by sex (n = 595 men, 637 women). Note: Figures showing trends include only men and women in the analytical sample, so values may differ slightly from those shown in Table 1.

**Fig 4:**
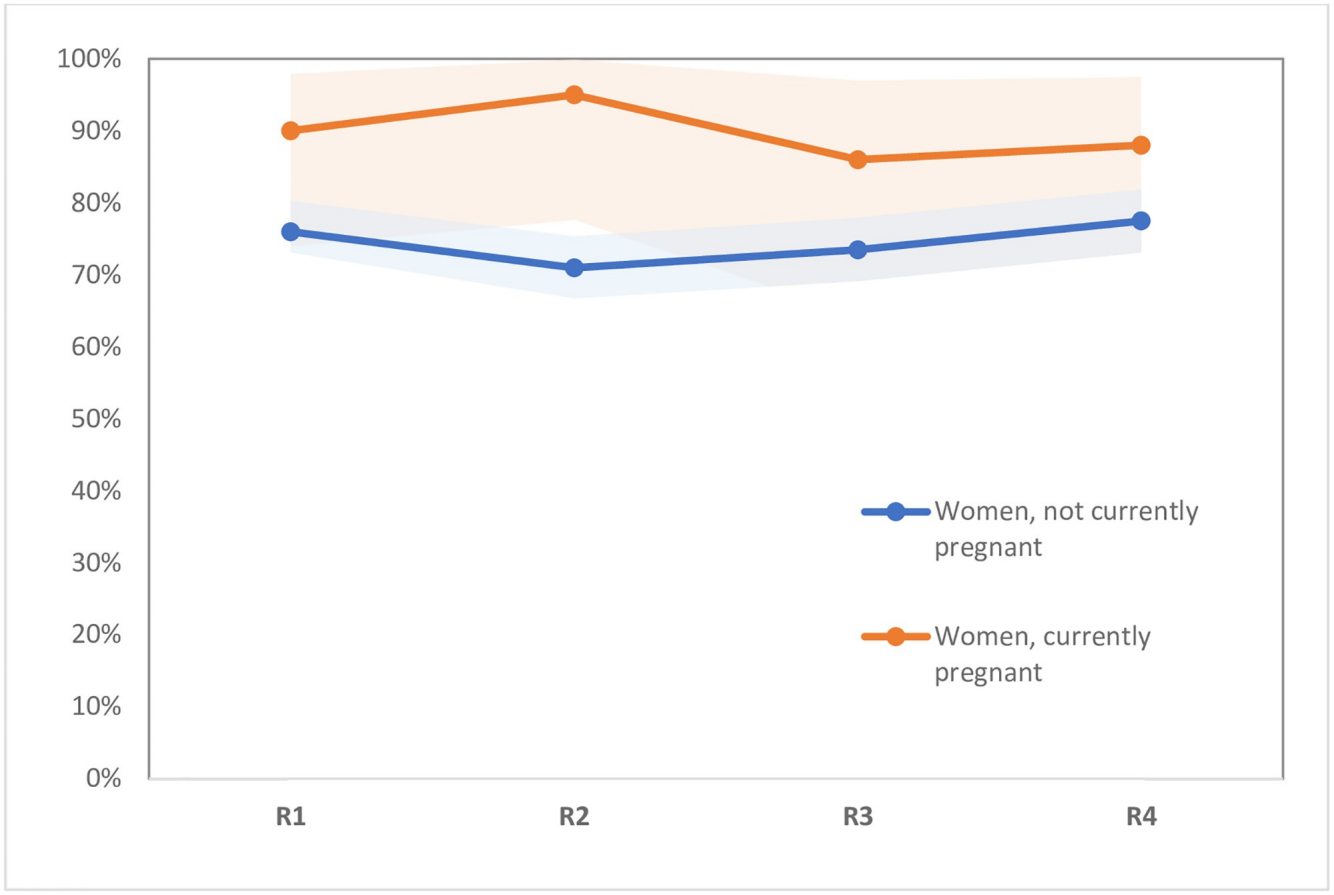
Percent women reporting that they were tested for HIV in the last 6 months, and 95% confidence intervals, by current pregnancy status (n = 481 non-pregnant women and 43 pregnant women at baseline). Note: Figures showing trends include only women in the analytical sample, so values may differ slightly from those shown in Table 1.

**Fig 5:**
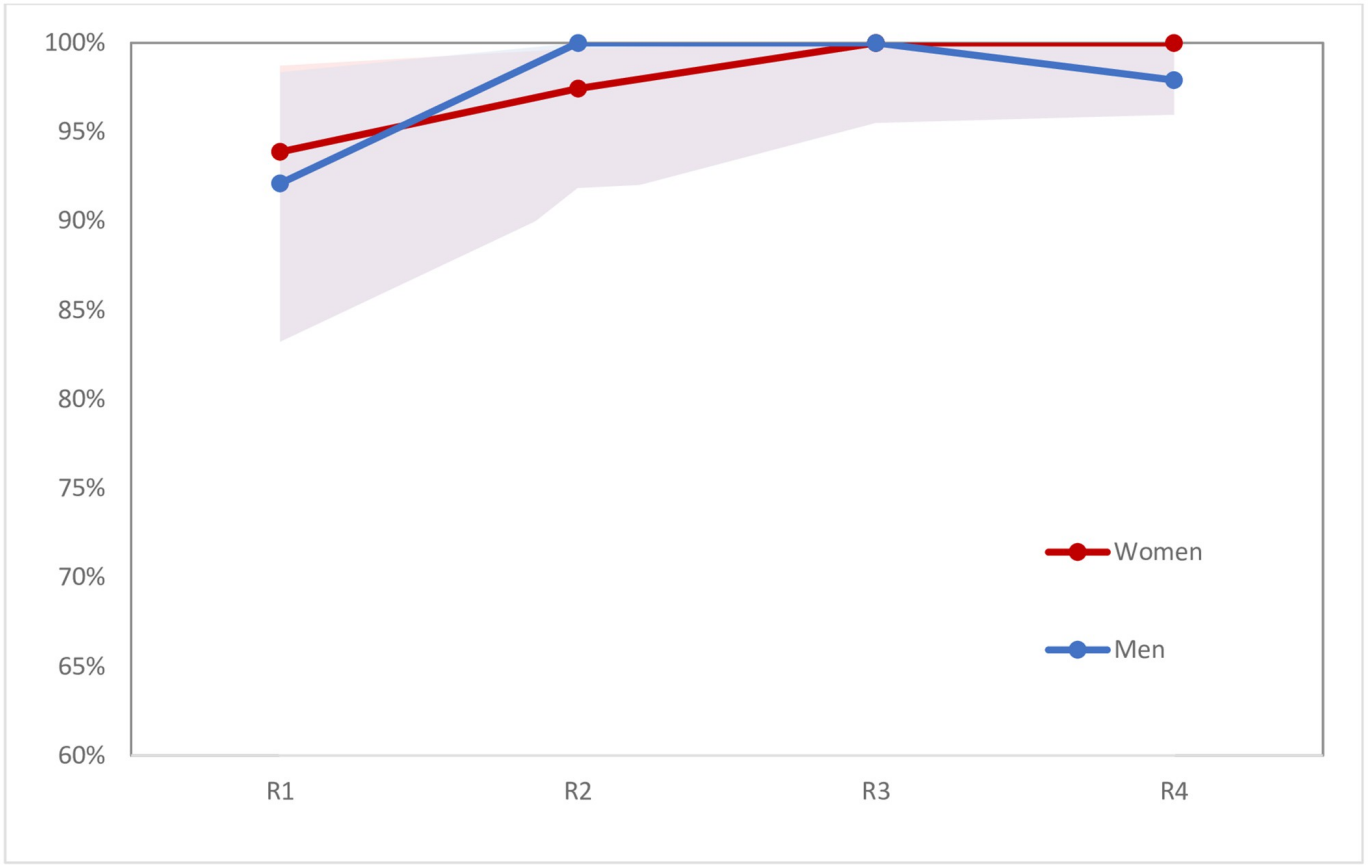
Percent currently on antiretroviral treatment, among those reporting HIV-positive status, and 95% confidence intervals, by sex (n = 43 men, 57 women at baseline). Note: Figures showing trends include only men and women in the analytical sample, so values may differ slightly from those shown in Table 1.

During follow-up, reported HIV prevalence increased among women from 14.3% to 18.8% (p<0.0001), and among men from 8.7% to 11.8% (p<0.01) (see Fig 2). In both the full baseline and analytical samples (i.e., those interviewed at least twice), 14.3 percent of women reported being HIV-positive, whereas self-reported HIV prevalence among men was 7.6% in the full baseline sample and 8.7% in the analytical sample (p = 0.029). HIV testing levels remained relatively stable throughout follow-up among women in the analytical sample, with 77% reporting having been tested in the last six months at baseline, and 78% at the final round of follow-up (see Fig 3). However, these testing levels varied by pregnancy status. Testing in the last six months declined slightly for non-pregnant women, from 76% at baseline to 73% in the final round (p<0.05), while remaining high for currently pregnant women across rounds (ranging from 86% to 95%) (see Fig 4). Similarly, levels of testing in the last six months among men who did not know their status declined slightly, from 67% at baseline to 63% in the final round (p<0.05) (see Fig 3). Among those who reported being HIV-positive at baseline, ART use in the last six months was high at baseline (95% for HIV-positive men and 96% of HIV-positive women). These levels increased to nearly 100% in both groups by the last round of follow-up, but the samples included were small, especially for men (see Fig 5).

Given the high levels of treatment at baseline (and small samples of men and women reporting their HIV-positive status), we are unable to assess the effects of the CR program on use of treatment in the last six months among those who reported being HIV-positive. However, we were able to assess whether participation in the CR program was associated with increased testing for women and men (see Table 3). Both random effects and fixed effects models for women indicated that the probability of having been tested for HIV in the last six months was 17% higher for women who participated in the program, compared to those who did not participate. As a robustness check, we removed currently pregnant women from the model since it was likely that their high levels of testing were due to exposure to antenatal care. However, the results for women were unchanged in those models (results not shown). For men, we found no association between program participation and levels of testing in random effects or fixed effects models.

**Table 3:** Estimated effects of the Community Responses Program on the likelihood of reporting having been tested for HIV in last 6 months, by sex.

	Women (n = 521)				Men (n = 510)			
	Random Effects		Fixed Effects		Random Effects		Fixed Effects	
Program participation (Stepping Stones)	1.17	*	1.17	*	1.06		1.05	
Program participation (other)	0.94		0.93		0.96		0.98	
Rollout phase	1.01		1.32	*	1.00		0.96	
Time since last interview (months)	1.00	~	1.00	~	0.99	**	0.99	**
Follow-up round	1.01		1.00		1.00		0.99	
*Baseline characteristics and behaviors*								
Age	0.99	~	--		1.00		--	
Assets	0.99		--		1.00		--	
Education (ref = less than grade 12)	1.02		--		1.01		--	
Employment (ref = unemployed)								
Employed	0.99		--		0.99		--	
Social grants/family support	0.99		--		0.98		--	
Time lived in community (ref = 0–12 months)								
13–24 months	0.96		--		0.98		--	
25+ months	0.96		--		1.03		--	
Tested for HIV in last six months	1.48	***	--		1.57	***		
Sex with main partner in last 6 months	1.06	*	--		0.99			
Currently pregnant	1.06		--					
Observations	1625		1625		1621		1621	
Groups	521		521		510		510	

***Notes***: Estimates are from linear random effects and fixed effects regression models, which control for clustering at the community level and repeated observations. Models exclude those who reported they were HIV-positive in the previous round. Fixed effects models also control for individual-level unobserved factors that remain stable over time. We run linear rather than logistic regression models to maintain the sample of respondents who experienced no change in the outcome over time (Allison 2009). All models include the analytical sample, which is those who participated in at least one follow-up round of data collection.

#### What drives HIV testing for women and men?

For both men and women in the study communities, once they test positive for HIV, accessing treatment was nearly universal by endline. Yet these results highlight a remaining gap in service delivery related to testing, especially for men. In order to identify possible opportunities to close this gap, we explored the drivers of testing for both men and women in this study.

We began by trying to understand whether there were certain groups who were most in need of testing, or whether overall testing was occurring less frequently than every six months for most men and women. To explore this question, we divided the analytical sample into groups based on how frequently they reported during follow-up that they had been tested in the past six months. We found that, during follow-up, 20% of men and 9% of women were not tested at all (the “never testers”). And 25% of men and 36% of women reported they were tested in 3 or 4 rounds (the “frequent testers”), or about every six months. The middle group, who reported they were tested 1–2 times during follow-up, was the same size for men and women: 55%.

We then tried to understand the characteristics of men and women who were tested during follow-up (that is, in either the middle group or frequent testers group) (See Table 4). For men, those who migrated recently to the community were less likely to be tested. Xhosa men were also less likely than Zulu men to test during follow-up; however, on average Zulu men had lived in the community for longer than Xhosa men, which may explain this difference. On the other hand, men who slept away from their home for more than 30 days in the last 6 months were more likely to get tested (88%) than men who did not sleep away from their home (79%). For women, we found no differences in the proportion testing during follow-up by these baseline characteristics. In the analytical models for men, the strongest (and only clear) predictor of having been tested in the last 6 months during follow-up was whether they had been tested in the last six months at baseline. Baseline testing was also significantly associated with testing during follow-up for women.

**Table 4:** Proportion who ever tested for HIV during follow-up, by baseline characteristics and sex.

	Men		Women
	(n = 760)		(n = 768)
*Length of time living in community*		**	
0–12 months	70.6%		90.0%
13–24 months	85.9%		91.6%
25+ months	82.1%		91.3%
*Slept away from home > 30 days (past 6 months)*		***	
Yes	88.3%		90.1%
No	78.6%		91.0%
*Ethnic group*		*	
Zulu	81.4%		90.9%
Xhosa	74.2%		91.8%
Other	100.0%		91.7%
*Primary source of income (past 3 mos)*		~	
Unemployed, no income	86.0%		92.6%
Employed	83.5%		93.4%
Social grants/Family	74.1%		94.0%

***p<0.001;

**p<0.01;

*p<0.05,

^~^p<0.10

Third, we explored whether “never testers” participated in Stepping Stones during follow-up. At endline for men, 8% of those who had participated in the program were “never testers” and 22% of non-participants were “never testers” (p<0.0001). For women this difference was smaller: 5% of participants were “never testers” compared to 10% of non-participants (p = 0.08).

## Discussion

This study identified some promising patterns of HIV service utilization by adults living in informal settlements in KZN. The vast majority (90%) of both men and women had ever been tested for HIV at baseline, but a much smaller proportion (25% of men and 36% of women) reported being tested about every six months during follow-up. Given continued high risk of HIV experienced by many men and women living in informal settlements, regular testing is essential to meet the UNAIDS testing and treatment and viral suppression goals (i.e., the ‘90:90:90’ goals). Despite high levels of testing in the last six months for pregnant women (around 90% in each round), testing levels were lower for non-pregnant women (78% at endline in 2019) and decreased over time for men (to 63% at endline in 2019). Yet these levels compare favorably to data collected in KwaZulu-Natal in 2015, which found that 42% of women and 35% of men of all ages had been tested for HIV *in the previous 12 months* [25]. The higher levels of testing reported in our study likely reflect general trends of increasing testing levels in the province over the last five years. Perhaps most promising, by the end of follow-up nearly all HIV-positive study participants reported that they were on treatment. These levels are substantially higher than those reported in 2015 in KZN, which ranged from 60% (ages 20–24) to 74% (ages 25–29) for women and 71% (ages 25–29) to 79% (ages 35–39), for men in comparable age groups [25]. The findings from this study are generalizable to the larger population of those living in informal settlements in KwaZulu-Natal due to our sampling approach, and comparability of our findings to those of a recent study conducted in 34 other informal settlements in eThekwini [26, 27].

Consistent with previous evidence from South Africa [28], we found that reaching men with testing services remains challenging [28]. Recent qualitative research found that illness was the primary driver of testing for HIV-positive men in South Africa, Malawi, Eswatini and to a lesser extent Uganda [29]. Consistent with our findings, men in each setting reported that they were rapidly linked to HIV care after receiving a positive test result. Another recent analysis from research in the same countries sought to unpack the experiences of HIV testing for men and identified three groups of testers: ‘vigilant’ testers, who test regularly, ‘opportunistic’ testers, who test when presented with an opportunity through programming, and ‘resistant’ testers, who are unlikely to test even when the opportunity is presented [30]. Resistant testers mainly saw negative outcomes of becoming aware of their HIV status (e.g., stigma and dissolution of relationships if they were to be diagnosed with HIV; not being able to continue work). However, some men (particularly in Uganda) described remarkable vigilance about testing with each partner, and at multiple key points of the relationship (e.g., when wishing to stop using condoms or to live together / get married) [31].

Building on that work, we sought to identify the group of men and women in our study who were most resistant to testing, and to describe their characteristics in order to inform future programming efforts. Previously documented barriers to male engagement in HIV services such as testing include a number of social and structural factors [32]. For example, HIV-related stigma and fear of disclosure leads to delays in HIV testing [33]. A lack of awareness about the benefits of early diagnosis and treatment can keep people who are living with HIV out of care. Common gender norms may also discourage men from engaging in testing. Rigid constructions of masculinity and male gender norms associated with toughness and control, sexual prowess, as a way of asserting manhood can deter men from engaging with HIV services [34].

Structurally, living in informal settlements may make it difficult for men to connect to existing health systems [7]. We found some evidence of these patterns; men who were newer to the community were more likely to be in the “never testers” group than those who had lived in the community for a year or more, and perhaps had deeper community ties. Other recent work in informal settlements in KZN found that young men and women were more likely to regularly attend a similar community-based intervention (i.e., a version of Stepping Stones) if they had lived in the community for longer [35]. These findings underline the importance of community ties in engaging men and women in programs and services. On the other hand, we found that men who had slept away from home for more than 30 days in the past six months were more likely to be tested than those who had not, perhaps indicating better linkage to service in other settings, or riskier sexual behavior while away from home that leads to more testing (although we do not observe this in the data).

Programs designed to increase levels of HIV testing should ensure they reach the populations most in need. Although participation in Stepping Stones was associated with higher levels of testing for women in our study, we observed no relationship between program participation and testing for men. We found evidence that those who did not get tested during follow-up were also less likely to have participated in the Community Responses program. This finding could reflect two underlying patterns: either participation in the program led those who started off as “never testers” to start testing. Or “never testers” were less likely to decide to participate in the program. For men, the latter pattern seems to hold, since we find no evidence of an association between program participation and an increase in testing. For women, the similar proportion of “never testers” in the participant and non-participant groups is consistent with the fact that program participation was associated with testing for women. In sum, while there is some evidence that the program led to more testing for women, it did not appear to lead to more testing for men, perhaps because the program did not reach those most in need of testing.

We did not find many significant differences between program participants and non-participants based on demographic characteristics, indicating that these characteristics may not clearly predict the likelihood of testing. We also found no significant differences between the results from random effects and fixed effects models, suggesting that significant program effects for women are unlikely to be explained by selectivity of program participation based on characteristics such as education level or ethnic group [24]. Rather, in a highly mobile population experiencing economic insecurity, tendency to test might change as circumstances, such as employment opportunities, evolve.

As with any program evaluation, there are several limitations to this study. First, given departures from the original stepped wedge design, we shifted our original analysis plans to instead focus on the association between participation in the program and our outcomes of interest. Baseline data indicate that those who participated and did not participate in program activities were largely similar based on demographic characteristics. However, they were not similar based on key behavioral characteristics, including history of testing. Further, our data show that the men reached by the CR program were less likely to be tested for HIV than those who were not reached. If the same pattern holds for the study population, we may be underestimating challenges in reaching men with testing services.

Near universal HIV treatment for those who tested positive for HIV demonstrates encouraging trends in access to HIV services in these communities. Yet barriers remain to testing, especially for men. The low rates of HIV testing among men—in South Africa and other settings—has garnered much attention over the last few years. There is a recent proliferation of projects to try to address this challenge in South Africa, and much will be learned [36]. Emerging research can point programmers in the right direction by creating HIV risk profiles of men to understand groupings of characteristics that lead to less or more HIV testing [37]. This study offers insights into which men and women are least likely to be tested regularly, and whether programs are effectively reaching those groups. Building the global evidence base on these questions is vital to improving outcomes related to HIV prevention and care.

## Supporting information

S1 Checklist CONSORT 2010 checklist of information to include when reporting a randomised trial. (DOC)

S1 File Evaluation of the PEPFAR/USAID Community Responses Program among adults in informal settlements in KwaZulu-Natal, South Africa. (DOCX)
