# Supplementary material for: What are we learning about HIV testing in informal settlements in KwaZulu-Natal, South Africa? Results from a randomized controlled trial
Source: PLoS One. 2022 Mar 8;17(3):e0257033. doi: 10.1371/journal.pone.0257033 (PMC8903271; doi:10.1371/journal.pone.0257033)
Supplement: S1 File — (DOCX) [file pone.0257033.s002.docx]

**Project title:** Evaluation of the PEPFAR/USAID Community Responses Program among Adults in Informal Settlements in KwaZulu-Natal, South Africa

**Principal Investigators:**

**Dr. Stephanie Psaki**

Population Council

New York, NY

+1-212-339-0691

spsaki@popcouncil.org

**Dr. Paul C. Hewett**

Population Council
Washington DC

+1-202-937-9419
phewett@popcouncil.org

**Dr. Mags Beskinska**

MatCH Research Unit

University of the Witswaterstrand

Durban, South Africa

+27-71-476-5174

mbeksinska@matchresearch.co.za

**Dr. Julie Pulerwitz**

Population Council,
Washington DC

+1-202-937-9436

[jpulerwitz@popcouncil.org](mailto:jpulerwitz@popcouncil.org)

**Co-Investigator :** **Letitia Greener**

MatCH Research Unit

University of the Witswaterstrand

Durban, South Africa

[lgreener@matchresearch.co.za](mailto:lgreener@matchresearch.co.za)

**Study Coordinator :** **Brady Zieman**

Population Council,
Washington DC

+1-202-937-9436
bzieman@popcouncil.org

**Project location:** KwaZulu Natal, South Africa

**Proposed project dates:** **October 1, 2015 – August 14, 2019***October 1, 2015–March 31, 2016 (proposal development)*
*April 1, 2016 – August 14, 2019 (project term)*

**Submitted to the IRB:** April 28, 2016

**Paul C. Hewett**  28 April, 2016

This work will be conducted under Project SOAR (Cooperative agreement AID-OAA-A-14-00060), made possible by the generous support of the American people through the United States President’s Emergency Plan for AIDS Relief (PEPFAR) and United States Agency for International Development (USAID). The contents of this protocol are the sole responsibility of the authors and do not necessarily reflect the views of PEPFAR, USAID, or the United States Government.

Table of Contents

[Summary of Proposed Research 1](#_Toc449618966)

[Background and Rationale 1](#_Toc449618967)

[Addressing social and structural barriers that increase vulnerability to HIV 2](#_Toc449618968)

[Preventing new HIV infections 2](#_Toc449618969)

[Expected results 2](#_Toc449618970)

[Intervention Description 4](#_Toc449618971)

[The Community Responses (CR) program 4](#_Toc449618972)

[Research and Evaluation 6](#_Toc449618973)

[Goals and objectives 6](#_Toc449618974)

[Overview of research activities 7](#_Toc449618975)

[Impact evaluation 9](#_Toc449618976)

[Informed consent process for surveys 17](#_Toc449618977)

[Routine program monitoring data 20](#_Toc449618978)

[Implementation Science (IS) 21](#_Toc449618979)

[1. IDIs and FGDs with cohort participants 22](#_Toc449618980)

[2. IDIs and FGDs with Community Responses staff 24](#_Toc449618981)

[3. Session Observations 26](#_Toc449618982)

[Qualitative Data Analysis 27](#_Toc449618983)

[Data Management 27](#_Toc449618984)

[Work Plan 28](#_Toc449618985)

[Research utilization strategy 28](#_Toc449618986)

[Capacity strengthening activities 28](#_Toc449618987)

[Personnel and implementing organizations 28](#_Toc449618988)

[Products 29](#_Toc449618989)

[References 31](#_Toc449618990)

[ANNEX A: Adult Survey Informed Consent 33](#_Toc449618991)

[ANNEX B: Participant In-Depth Interview Informed Consent 38](#_Toc449618992)

[ANNEX C: Participant Focus Group Discussion Informed Consent 42](#_Toc449618993)

[ANNEX D: Program Manager In-Depth Interview Informed Consent 46](#_Toc449618994)

[ANNEX E: Program Staff Focus Group Discussion Informed Consent 50](#_Toc449618995)

[ANNEX F: Survey Questionnaire 54](#_Toc449618996)

[ANNEX G: In-depth interview guide: Stepping Stones participants 101](#_Toc449618997)

[ANNEX H: Focus group discussion guide: Stepping Stones participants 105](#_Toc449618998)

[ANNEX I: In-depth interview guide: Stepping Stones program managers 109](#_Toc449618999)

[ANNEX J: Focus group discussion guide: Stepping Stones staff and facilitators 113](#_Toc449619000)

[ANNEX K: Study information Sheets 117](#_Toc449619001)

[ANNEX L: Research Ethics Training Certificates 127](#_Toc449619002)

# Summary of Proposed Research

The primary goal of the proposed Community Responses (CR) program evaluation will be to determine the extent to which the United States Agency for International Development (USAID)/South Africa-funded CR program (and particularly the effects of community-based HIV/gender-based violence (GBV) prevention programming, which include a component to create demand for related services) is effective at reducing HIV and sexual and GBV risk, and improving related service utilization. Key outcomes to be measured include:

- Increased uptake of HIV and GBV services [e.g., HIV testing and counseling (HTC), sexual and gender-based violence (SGBV) support services]
- Improvements in sexual risk behaviors
- Reduction in experience/perpetration of SGBV
- Decreased support for harmful gender norms

Project SOAR proposes a cluster randomized stepped-wedge evaluation design, in which clusters are randomized to when they initiate the CR intervention. A cohort of adult men aged 18–35 years and women aged 18–24 years in the evaluation communities will be followed over the course of approximately 32 months, with a baseline survey and three follow-up interviews at approximately ten month intervals. The evaluation will be conducted in selected informal settlements in eThekwini and Ugu in KwaZulu-Natal Province, one of four provinces where the CR program is being implemented.

We will supplement this information with an analysis of District Health Information System (DHIS) data, indicating trends in service utilization, and risk/prevention behaviors, where feasible.

In addition, we will conduct implementation science research to explore the experience of updating and scaling up the Stepping Stones intervention for men and women in informal settlements. This will include qualitative interviews with program implementers, and a selection of study participants.

Overall, this study offers the opportunity to contribute to several critical domains of interest for PEPFAR and the wider HIV and AIDS community. It will assess the effectiveness of a community-based HIV prevention program designed to promote uptake of HIV and SGBV services and transform gender norms to create an enabling environment for HIV prevention behaviors among adult men and women. Building the global evidence base on these questions is vital to both improved outcomes related to HIV prevention and eliminating harmful gender norms, which are linked to a range of other outcomes of concern. These findings will be particularly relevant for the South African context, given the generalized epidemic setting and high levels of reported GBV.

# Background and Rationale

South Africa’s National Strategic Plan (NSP) on HIV and AIDS, sexually transmitted infections (STIs), and tuberculosis (TB) for 2012–2016 sets out four strategic objectives: (1) addressing social and structural barriers that increase vulnerability to HIV, STI, and TB infections; (2) preventing new HIV, TB, and STI infections; (3) sustaining health and wellness; and (4) increasing the protection of human rights and improving access to justice (South African National AIDS Council 2011). The CR program aims to address the first two objectives.

## Addressing social and structural barriers that increase vulnerability to HIV

Gender norms are associated with a host of sexual and reproductive health outcomes and risk behaviors, such as condom and contraceptive use, even after controlling for other factors (Tang et al. 2001; Stephenson et al. 2012; Karim et al. 2003). Gender norms are also closely tied to other antecedents of HIV infection, especially intimate partner violence (IPV) and power in sexual relationships. Inequitable gender norms and traditional masculinity norms have been associated with female IPV victimization (Gomez et al. 2011), and male perpetration of IPV or rape (Gomez et al. 2011; Shannon et al. 2012). The consistency of such findings—across countries, continents, class, and race—is striking. A growing body of evidence suggests that programs that seek to change harmful gender norms can improve health behaviors and outcomes such as number of sex partners, consistency of condom use, STIs, and IPV (Jewkes et al. 2008; Pulerwitz et al. 2015). These programs often focus on convening small groups of participants and engaging them in learner-centered and interactive activities that foster critical reflection about gender norms and inequality, and may complement these with broader behavior change communication activities (Dworkin et al. 2013; Pulerwitz et al. 2010; Pulerwitz et al. 2012).

## Preventing new HIV infections

Utilization of HIV testing services, and linkages to care and treatment for those who test positive, are of critical interest, both because of the promise these hold for reducing HIV transmission and for connecting consumers to the broader health system to improve their treatment outcomes. In the most recent South African National HIV Prevalence, Incidence and Behaviour Survey (Shisana et al. 2014), more than 90% of the population reported knowing where to get an HIV test, while only 65% had ever been tested. In fact, national survey data demonstrated that 62% of men and 45% of women testing positive were unaware of their infection status. Increasing the proportion of the population who are aware of their status can have an immediate impact on treatment uptake and prevention of new infections (Shisana et al 2014).

## Expected results

This study offers the opportunity to contribute to several critical domains of interest for PEPFAR and the wider HIV and AIDS community, as well as addressing HIV program challenges of great relevance for the South Africa context. It will assess the effectiveness of a community-based HIV prevention program designed to promote uptake of HIV and SGBV services and transform gender norms to create an enabling environment for HIV prevention behaviors among adult men and women. The study will also contribute to important questions of how much intervention (dose) is needed to achieve key outcomes.

Further, this research will provide evidence on the experience of implementing a scaled-up version of Stepping Stones in informal settlements (implementation science). There are many outstanding questions about how to best roll-out/scale up this intervention, which has demonstrated evidence of impact in controlled environments. In this case, Stepping Stones is being applied with a reduced number of sessions (than those rigorously evaluated in the past, to permit more wide spread roll out), and with implementing partners that have not worked much with the intervention before.

Building the global evidence base on these questions is vital to both improved outcomes related to HIV prevention and eliminating harmful gender norms, which are linked to a range of other outcomes of concern. These findings will be particularly relevant for the South African context, given the generalized epidemic setting and high levels of reported gender-based violence.

# Intervention Description

## The Community Responses (CR) program

The United States Agency for International Development (USAID)/South Africa is supporting the five-year Community Responses (CR) program (2014-2019), implemented by the Centre for Communication Impact (CCI). The target population for the CR program is young men (ages 15–35) and women (ages 15–24)^[[1]](#footnote-2)^ living in informal settlements in the United States President’s Emergency Plan for AIDS Relief (PEPFAR) communities, located in 23 sub-districts within the Gauteng, Kwazulu-Natal, Western Cape, and Mpumalanga provinces.

The CR program includes the following reinforcing components:

1. *Standardized HIV prevention interventions* (PP_PREV),^[[2]](#footnote-3)^ defined as interventions that are designed for a specific priority population to reduce HIV transmission, implemented the same way each time, adhere to written protocols, include goals and activities tailored to the priority population, comprise multiple encounters with the same individuals or small groups, and have a system for tracking and reporting the completion of every element of the intervention. Specific interventions include ZAZI^[[3]](#footnote-4)^ and Brothers for Life (1x2 hour sessions).^[[4]](#footnote-5)^ Participants will be encouraged and referred for services including: HIV testing and counseling (HTC), voluntary male medical circumcision (VMMC), prevention of mother to child transmission of HIV (PMTCT), contraception, condoms, antiretrovirals (ARVs); uptake and adherence), and sexual and gender-based violence (SGBV).
2. *Structural interventions (SI) for gender norms for HIV and gender-based violence (GBV) prevention* (GEND_NORM),^[[5]](#footnote-6)^ are defined as interventions that support participants to understand and question existing gender norms and reflect on the impact of those norms on their lives and communities; make a clear link between the gender norms being discussed and HIV prevention, treatment, care or support; and have a minimum of 10 hours contact time per individual. Specific interventions will include grassroots soccer/SKILLZ curriculum^[[6]](#footnote-7)^ (in-school youth aged 15–19), and Stepping Stones for youth and adults aged 20 and older.^[[7]](#footnote-8)^ Participants will be encouraged and referred for services including: HTC, VMMC, PMTCT, contraception, condoms, ARVs (uptake and adherence), and SGBV and post-exposure prophylaxis (PEP) services.
3. *SGBV prevention activities:* These are defined as interventions that raise awareness of SGBV and increase knowledge and access to PEP; promote and refer to local SGBV service providers, where they exist; refer to the South African Police Services, psychosocial support services, and relevant government departments; and emphasise networks, key stakeholders, and service providers to ensure joint implementation and effective referrals. Specific interventions will include: community dialogues focused on SGBV prevention and workshops for men (10x1 hour sessions).
4. *Community dialogues*: The CR program will also include situational analyses and mapping activities before interventions begin, and will provide feedback on community achievements after interventions. Community dialogues will take place in all CR communities.

The CR program was designed to align with the Government of South Africa’s National Strategic Plan for HIV, sexually and transmitted infections (STIs), and tuberculosis (TB) (NSP) for 2012–2016, as well as with the UNAIDS targets for 2020: 90% of people living with HIV knowing their status, 90% of those who know their status on ART, and 90% of those on antiretroviral treatment (ART) virally suppressed (90:90:90 target).^^[[8]](#footnote-9)^^

# Research and Evaluation

## Goals and objectives

The overall goal of this research is to generate and disseminate evidence regarding community-based interventions aiming to prevent HIV transmission and reduce GBV (and increase HIV/GBV service use) among adult men and women in a high prevalence setting. Specifically, the CR evaluation has the following primary and secondary objectives:

***Primary objective:*** Conduct an **impact evaluation to** assess the effect of the CR program on key outcomes for adult men and women living in communities exposed to the CR program, compared to those living in control communities, on key program outcomes.

- The primary analyses, based on an intent-to-treat approach, will compare at the community or cluster level the average change in outcomes after full CR program exposure to the average change in outcomes in communities that have not yet received the CR intervention.
- Additional analyses will also assess whether individual or community-level program exposure (e.g., proportion of the community that participated in each component) leads to improvements in key program outcomes on an as-treated basis.^^[[9]](#footnote-10)^^
- Further, analyses of district health information software (DHIS) data from health facilities in program catchment areas will also be used to assess trends in service utilization for key HIV and SGBV services.

***Secondary objectives:*** Conduct **implementation science research** to assess participant perspectives and experiences with the Stepping Stones component of the CR program, as well as to provide a deeper understanding of the implementation process and reasons for program effectiveness. As part of this effort, we will conduct:

- Qualitative analysis of in-depth interviews (IDIs) and focus group discussions (FGDs) with a subset of program participants to learn about their experiences and recommendations regarding the program.
- Qualitative analysis of IDIs and FGDs with program staff to discuss the process, experiences, challenges, recommendations, and benefits of implementing (and scaling up) the current version of Stepping Stones, as well as updating information related to current treatment (and treatment as prevention) guidelines.
- Direct observations of a limited selection of Stepping Stones meetings to assess the fidelity of program implementation to program design, the ability of the facilitators to convey core information and to foster interactive sessions, and the responsiveness of the participants to the material and sessions.

## Overview of research activities

An overview of the research activities, methods, and study instruments is provided in Table 1, with a discussion of each study component following. There are two broad research components: 1) an impact evaluation of the CR program and, 2) implementation sciences to provide insights to how the intervention was received among the target population and how to improve its effectiveness if it was to be scaled up in other settings.

**Table 1: Overview of research activities and methods**

| **Study Component** | **Impact Evaluation** | | **Implementation Science** | | |
| --- | --- | --- | --- | --- | --- |
| **Research Activity** | 1. **Stepped-wedge cluster randomized trial** | 1. **Extraction of routine program monitoring data** | 1. **In-depth interviews (IDIs) and focus group discussions (FGDs) with cohort participants** | 1. **IDIs and FGDs with Community Responses staff** | 1. **Observations of Stepping Stones Sessions** |
| **Study**  **population** | Panel of women (aged 18–24) and men (aged 18–35) living in selected communities | CCI monitoring data | Selected panel study participants:  women (ages 18–24) and men (ages 18–35) | Facilitators, staff, and program managers involved with Community Responses | Participants and facilitators in stepping stones sessions |
| **Sample size** | 1,500 participants:  750 women  750 men | Not applicable | 60 IDIs : 15 women, 15 men  6 FGDs: 2x 10 women  2x 10 men  2x 5 women/5 men | 10 IDIs : Program managers  4 FGDs: Program facilitators  and staff | Observation of approximately 5 – 10 sessions |
| **Location of**  **activity** | Ugu and eThekwini sub-districts, KwaZulu Natal, South Africa | Ugu and eThekwini sub-districts, KwaZulu Natal, South Africa | Ugu and eThekwini sub-districts, KwaZulu Natal, South Africa | Ugu and eThekwini sub-districts, KwaZulu Natal, South Africa | Ugu and eThekwini sub-districts, KwaZulu Natal, South Africa |
| **Timing** | Stepped-wedge intervention roll-out with 4 survey rounds (2016–2019):  *(See Table 2)* | Throughout follow-up period  (2016–2019) | After stepped-wedge intervention rollout (variable) and again at endline (Feb 2019) | After stepped-wedge intervention rollout (variable) and again at endline (Feb 2019) | During follow-up period  (2016–2019) |
| **Method** | Structured interviewer‐administered  interview | Record extraction | IDI & FGD | IDI & FGD | Unstructured observation |
| **Informed**  **Consent** | Adult (Annex A) | Not applicable: data aggregated at community level | Participant IDI (Annex B)  Participant FGD (Annex C) | Staff IDI (Annex D)  Staff FGD (Annex E) | Routine monitoring visits |
| **Study**  **Instrument** | Questionnaire  (Annex F) | Extraction tool | Participant IDI guide  (Annex G)  Participant FGD Guide (Annex H) | Staff IDI guide  (Annex I)  Staff FGD Guide (Annex J) | Observation notes |

## Impact evaluation

A **stepped-wedge cluster randomized trial** with an embedded prospective cohort sample is proposed to provide rigorous evidence of the effects of the full CR program. Through a stepped-wedge evaluation design, clusters (communities) cross over from control to intervention at different time points (Hussey & Hughes 2007; Brown & Lilford 2006). A community is defined as an informal settlement as this is the locus of the CR intervention. Rather than clusters being randomized to control or intervention groups for the study duration, the time at which each cluster crosses over from control to intervention is randomized (Hussey & Hughes 2007). A 2006 systematic review of published studies using stepped-wedge designs found that they were most commonly used in HIV treatment and prevention programs in developing countries (Hughes et al. 2003; Brown & Lilford 2006). The stepped-wedge evaluation design has been shown to be particularly useful for testing community-level interventions previously shown to be effective at the individual level (Hussey & Hughes 2007).

The stepped-wedge evaluation will be implemented in a subset of communities within the broader CR program implementation. In this context, the evaluation will not impede the general rollout of the CR program in non-evaluation areas. The proposed stepped-wedge design addresses several important challenges to constructing a control/comparison group in a widely implemented program such as CR: (1) CR program activities will necessarily have already begun in most targeted program communities before IRB approval is received for the evaluation, so that baseline data collection would not be possible in all CR communities without limiting the CR program; (2) communities where the CR program has not been targeted for implementation may participate in other activities during the same time period and therefore would not be appropriate control communities; and (3) the PEPFAR communities were not selected randomly (e.g., they may have higher HIV prevalence) implying that external communities may not be ideal control groups.

An illustrative schedule of the program and evaluation within a stepped-wedge approach is provided in Table 2. As is noted, a baseline survey (BL) for the cohort will be conducted in all sites at the study initiation for a period of approximately two months. Communities within the evaluation areas will be randomized to have the CR program rollout (R) in three steps at eight month intervals (Group A, Group B, and Group C), with continual implementation (I) after roll-out through the end of the program. The choice of three steps and eight month intervals for program roll-out is based on several considerations: the need to maximize the number of roll-outs (steps) in the evaluation period; the expected period of saturation of the intervention: and the expected period of program effects.^^[[10]](#footnote-11)^^ After each eight month program roll-out period, follow-up surveys (Round 2, Round 3, and Round 4) will be conducted among all study participants.

**Table 2. Illustrative schedule of program rollout and survey (32 months total)**

| **Beginning of period** | | **Aug 2016** | **Oct**  **2016** | **June 2017** | **Aug 2017** | **May 2018** | **June 2018** | **Feb 2019** |
| --- | --- | --- | --- | --- | --- | --- | --- | --- |
| Length of period (months) | | 2 | 8 | 2 | 8 | 2 | 8 | 2 |
| **Group A**  *(6 clusters)* | Survey | BL |  | R2 |  | R2 |  | R4 |
|  | Program rollout/ implementation |  | R | I | I | I | I | I |
| **Group B**  *(6 clusters)* | Survey | BL |  | R2 |  | R3 |  | R4 |
|  | Program rollout/ implementation |  |  |  | R | I | I | I |
| **Group C**  *(6 clusters)* | Survey | BL |  | R2 |  | R3 |  | R4 |
|  | Program rollout/ implementation |  |  |  |  |  | R | I |

**Notes**: In each time period, we assume two months for data collection followed by eight months for program implementation. “BL, R1, R2, R3, R4” indicates data collection; “R” indicates rapid program rollout after the respective survey has been completed; “I” indicates ongoing program implementation in the specified groups during the indicated time periods. Blank cells in the “program rollout/implementation” rows indicate that those communities will act as comparison groups during those periods.

There are several key requirements of this evaluation approach, which have important implications for implementation of the CR program:

1. *Randomized rollout of CR activities in evaluation communities*: CR activities will be rolled out randomly (at eight month intervals) within a pre-determined subset of CR communities. SOAR will work with CCI to randomize the schedule of rollout, and CCI will not initiate any program activities before rollout is scheduled for the community.
2. *Rapid rollout and saturation of all CR activities once program commences in each area*: The full package of CR activities will begin quickly throughout each community when that community’s implementation period begins. Also, the full package of CR activities should saturate each community quickly after the program commences in each cluster.
3. *Avoid contamination across clusters*: Clusters will be selected as to avoid contamination across clusters. Geographically separate clusters will be identified and the evaluation team will work with CCI to assure that the program does not cross-over into areas that are not yet scheduled for the intervention.

***Identification of study areas and eligible participants***

USAID and CCI have identified priority wards for the CR program within eThekwini and Ugu sub-districts in KwaZulu-Natal province, based on the prevalence of HIV in the area and the need for HIV prevention programming.^[[11]](#footnote-12)^ Within the identified wards, informal settlements were identified for targeting by the CR program. Depending on size, the informal settlements were further sub-divided into smaller clusters for the impact evaluation. A minimum number of households and clear demarcations (roads, highways, paths) were used as requirements for the area to by identified as a sampling cluster for the evaluation and subsequently stratified by high and low density areas. From the full list of approximately 80 informal settlements or sub-clusters, 18 were randomly selected proportional to size for the impact evaluation. After selection, clusters were than randomly ordered to receive the intervention at either the first, second, or third step as noted above in Table 1.

We propose to follow a cohort of 1,500 men and women living within each selected study cluster. Interviews will be conducted at baseline and three follow-up rounds (R2–R4). The Community Responses program as a whole aims to reach young men (aged 15–35), and young women (aged 15–24). The evaluation will be limited to men aged 18–35 and women aged 18–24. This is due to several reasons, including that: (1) the highest HIV incidence rates are found in this older age group, (2) the CR program intends to reach the younger group (aged 15–18) mainly through school-based programming, while this evaluation is particularly focusing on the effects of the community based work (including Stepping Stones, which is targeting people 20 and older), and (3) we expect that it would be quite difficult to recruit youth under 18 via the planned household-based sampling, as in-school youth would not often be found at home, and out of school 15–17 year old males are often be difficult to locate during daytime hours, when it is safer to conduct the surveys.

Eligible men and women will be sampled from households within the informal settlement. Households will be identified and sampled through the following means. All structures in the informal settlement will be manually numbered using Google Earth images and Google Earth GPS coordinates (longitude and latitude) will be assigned to the structure.^[[12]](#footnote-13)^ The structure listing will then be randomly ordered and interviewers will proceed through the list of numbered structures in the informal settlement in the order specified. A numbered map, GPS coordinates, landmarks, and distance measurements will be used to guide interviewers on the ground. If a structure is not a household it will be noted and the interviewer will proceed to the next structure on the list. A Kish grid will be used to select a household if there is more than one per structure.

One person in the eligible age group will be selected for participation per sampled household. Study interviewers wearing MatCH Research Unit identified T-shirts will approach the household and ask to speak to an adult/head of household. The interviewer will introduce themselves and offer an explanation of the study and survey and give an information sheet to the adult/head of household. They will also show a letter of permission from the ward councilor or other appropriate local gatekeeper. If the adult is willing, they will first be asked if there is at least one male or female living in the household who is within the eligible age group. If there is no individual who fulfils this criteria, the interviewer will thank the householder and proceed to the next house. If there is at least one person present who meets the study age requirements, the interviewer will continue and explain that in order to sample at the household level the fieldworker will need to list all household members (identified by age and initials only) using a household sample form (women aged 18–24, men 18–35) A Kish grid will be used to sample from the eligible household members if two or more individuals in the household meet the study inclusion criteria. If the selected individual is not in the household (working, shopping, etc.) the interviewer will ask to return at a convenient time to inform the household member about the study. The interviewer will return to the house on three different days at the preferred time in attempt to find the participant. If the participant cannot be reached after the third attempt, the interviewer will consider her/him unreachable.

Once the interviewer has obtained informed consent, the baseline survey (Annex F) will be conducted. Interviews will take place in a private location within the participant’s home. Participants will be able to choose an alternative community venue or MatCH Research offices if they do not wish to be interviewed at home. The interview will last approximately 60 minutes and will be conducted by an interviewer of the same sex as the participant. For the most sensitive questions around sexual and gender-based violence, the questions will be self-administered. Self-administration entails the participant reading the questions of the screening and entering their own responses directly without interviewer involvement. For follow-up interviews, staff will use the identifying and contact information collected at baseline to contact the participant, verify his/her identity, and conduct the follow-up survey (Annex F). If the study staff are unable to reach the participant at the follow-up interview, they will make two additional attempts on subsequent visits. Any participants who cannot be located for the follow-up interview will be noted as lost to follow-up.

Participants in the evaluation who have completed a survey may also be selected to receive a follow-up visit by CR program staff to provide more information about the CR intervention and invitation to events and/or programs that they may be interested in participating, e.g., Stepping Stones. The purpose of the outreach to survey participants is to assure sufficient exposure of study participants to the CR program and to provide a useful variable for the statistical modeling of impact (see section: Statistical analysis plan).

Specific eligibility criteria for inclusion in the evaluation are as follows:

- Between the ages of 18 and 24 (women) and 18 and 35 (men)
- Living in selected communities
- Can read in English or the local language
- Willing and able to give informed consent
- Willing to participate in three additional interviews at eight-month intervals
- Willing to provide research study staff with an identity number, address, phone number while participating in the study, and a fingerprint scan
- Agrees to participate in the study for the duration of up to three years; do not reasonably foresee moving out the study area within that time

***Primary outcomes and indicators***

Although a wide range of indicators will be measured and evaluated throughout the study period, a smaller representative set will serve as keystones for measuring program. These indicators are representative of the targeted outcomes sought by the CR program, including an increase us of HIV prevention and treatment services, reduction in sexual risk behaviors, reduction in the experience and perpetration of SGBV, and decreased support of harmful gender norms.

| **Outcome Domain** | **Indicator** |
| --- | --- |
| **Accessing HIV services** | % of participants who have obtained the results of an HIV test within the previous six months |
| **Accessing HIV services** | % of HIV-positive participants who have accessed HIV care and treatment services within the previous six months |
| **Sexual risk behaviors** | % of participants reporting using a condom at last sex |
| **Sexual risk behaviors** | % of participants reporting consistent condom use with a partner of unknown HIV status in the last 30 days |
| **SGBV** | % of participants reporting perpetrating or experiencing physical violence within the previous six months |
| **SGBV** | % of participants reporting perpetrating or experiencing sexual violence within the previous six months |
| **Gender norms** | % of participants who hold positive norms regarding gender based violence (from the GEM Scale) |
| **Gender norms** | % of participants who hold equitable norms regarding the roles of women and men (from the GEM Scale) |

**Power and sample size calculations**

To determine the required sample size of participants for the cohort, a Stata 13.0 sample size routine for stepped-wedge cluster randomized trials was used (Hemming and Girling 2014). The parameters specified for the sample size estimation include a study design with three implementation steps (of eight months in length), four total participant observations (baseline and one after each intervention step), a power criterion of 0.80, alpha coefficient of 0.05, and intra-cluster correlation of 0.05. A minimum 10% percentage point effect size (change due to intervention) was specified for the primary outcome indicators. As estimates of baseline prevalence for these indicators for the population of informal settlements by age is not readily available, the most conservative estimate of baseline prevalence (at 45%) was used. Sample size estimates were stratified by sex, such that the study is powered to draw inferences about impact separately for males and females; men and women would be sampled from different households. The minimal sample size required at the end of the study is approximately 1,260 individuals (630 men, 630 women), distributed across 18 clusters with 35 men and women in each clusters, 6 of which cross over from control to intervention at each of the three implementation steps. Assuming 15% loss to follow up, an estimated baseline sample size of 1,500 individuals (750 men, 750 women) has been specified.

**Statistical analysis plan**

The primary statistical comparison in a stepped-wedge design is between observations that have been exposed to the intervention and those that have not been exposed. In a stepped-wedge design cases will have periods of control (unexposed) and period of intervention (exposed). The first step in the analysis will be to evaluate whether the characteristics of the sampling clusters vary significantly by their original randomized implementation steps, i.e., the predetermined period of cross-over from control to intervention (Hemming et al. 2010). This analysis is conducted to assure that the original randomization did not produce early cross-over clusters that are inherently different with than later cross-over clusters. To assess balance in the clusters, analysis of community level and participant level indicators will be conducted across each of the randomized steps (Group A, Group B, and Group C from Table 2) using relatively simple statistical methods for assessing differences in grouped data, e.g., chi-square tests and t-tests.

The primary statistical approach to assessing the impact of the program will be an intent-to-treat (ITT) analysis where participants are analyzed according to their randomized period of exposure rather than whether they actually participated in the intervention at the time of implementation. While clusters are randomized to receive the CR program at a certain time, not all study participants will have actually been exposed the program when they were assigned to receive it, either because of a failure to saturate the program area or because individuals chose not to participate. The ITT analysis avoids such potential selection biases by using the exogenous random assignment of exposure as the indicator of impact. To estimate the impact within an ITT framework, generalized estimating equations (GEE) will be used that include random effects parameters for the cluster and for participants; the former to account for correlations of individuals with clusters and the latter to account for the repeated observation of participants in the cohort. Additional variables included in the regression will be calendar time, the group or period to which participants were randomized, and covariates that may play a role in the outcomes of interest (Heming et al. 2015; Hussey and Hughes 2007).

While the primary analysis and impact assessment will rely on an ITT approach, it will nonetheless be interesting to evaluate whether actual participation in the CR program predicts outcomes differentially if participation in the intervention is not 100%. It is important, however, to recognize the statistical estimation problems that result from selection into the program based on factors that can be/are measured and/or by unobservable factors. The central problem is that factors affect both participation in the program and outcomes and behaviors of interest leading to biased estimates of program impact if selection is not considered. To address these limitations, multiple estimation approaches will be explored. The first will be an instrumental variables regression, in which a model of program participation will be estimated using observed covariates and known exogenous variables, such as the original randomization period, and whether the participant was randomly chosen to receive program promotional material and outreach. A second approach will use fixed effects models that effectively control for the all unobserved constant influences from individual, family, and community characteristics.

## Informed consent process for surveys

After recruitment, the interviewer will complete four tasks during the informed consent process:

1. Explain the study and answer any questions
2. Screen for eligibility
3. Obtain written informed consent
4. Inform the participant that s/he may be invited to participate in an additional IDI or FGD.

If the selected resident is eligible, the interviewer will capture the participant’s identifying name, identification number, address, telephone number, secondary telephone number, email address, and fingerprint scan using a tablet computer or personal digital assistant (PDA). The research team will use this information to contact the participant for the three follow-up interviews that are planned during the three-year duration of the study. Personal identifying information for study participants stored in an encrypted, password-protected file, and only made available to relevant study personnel. A full description data security measures is included in the “Data Management” section.

Before administering the baseline questionnaire, the interviewer will briefly explain the study and its objectives to the selected participant. If the participant is still interested, the interviewer will either read aloud or ask the participant to read the appropriate informed consent document (Annex A). At this point, the interviewer will confirm that the participant expects to be available to participate in three additional follow-up interviews over the next three years, noting that the participant may also be invited to take part in an IDI or FGD. Finally, the interviewer will respond to any questions and concerns the participant may express about the study, emphasizing that participation is voluntary and that refusal to participate will affect neither the community standing nor services they may receive from the CR program, and that s/he may discontinue participation at any time.

Once all questions are answered to the participant’s satisfaction, the interviewer will ask the participant to provide written consent as an indication that they understand the risks and benefits of the study, and voluntarily agree to participate. If the participant is illiterate, the interviewer will ask the participant to identify a person who can sign the consent form on the participant’s behalf. To complete the informed consent process, the interviewer will sign the consent form and provide a copy to the participant.

No person will be enrolled until written informed consent is obtained. Informed consent will be obtained without coercion, undue influence, or misrepresentation of the potential benefits and risks that might be associated with study participation. All information will be presented in a language that will be easily understood by participants. The consent form will describe the risks and benefits associated with study participation. Participants will be informed that they can refuse to answer any questions, stop the interview at any time, or withdraw participation in the study. One copy of the signed consent form will be given to the participant, another original copy will be kept in a locked file in MatCH Research Unit’s office, separate from other source documents that would link study data with participant identification.

***Risks and benefits to participants***

*Risks:*

The primary risk associated with this study are possible breaches of participant confidentiality, as well as exposure to sensitive questions during the study interviews.

*Risks to confidentiality:*

Survey and interview content could include sensitive information that participants would not wish to be divulged to others. Data will be collected electronically, and every effort will be made to ensure that all study materials are stored securely. As this is a cohort study, data collectors will need to obtain contact information on all participants, but this information will be stored separately from collected data, and linking will be done through an identifying number. As detailed in the data management section, the research team will take all necessary precautions to ensure that participant contact information is never accidentally or intentionally made available to any non-privileged person.

*Additional risks:*

During the interviews, interviewers will ask sensitive questions about sex, HIV and other STIs, and GBV, which the participants could view as intrusive. As a result, some of the participants may become uncomfortable or distressed. The research team has designed the survey questionnaire to minimize unnecessary distress. The study team will pre-test the questionnaire among a small group of men and women to identify and eliminate unnecessary sources of discomfort or embarrassment. For the most sensitive questions around SGBV, the questions will be self-administered.

Similarly, the study team will train interviewers to be compassionate with participants, and to minimize their risk of distress. Experienced data collectors will conduct all study interviews, and interviewers will be instructed to stop questioning in cases where the participant shows signs of distress. The study will also establish a mechanism for referring distressed participants and victims of violence to a KwaZulu Natal public health facility for psychological counselling and survivor support services as needed.

*Benefits:*

No immediate tangible benefit is likely to accrue to the participants through their participation; this will be made clear when obtaining informed consent. However the interviewers will describe the potential benefits of conducting this study, for health care systems and clients thereof, so that prospective participants are fully aware of how the data gathered will be used to provide programmatic recommendations and help others in their community.

***Confidentiality***

Prior to participant recruitment, the study protocol, recruitment materials, and instruments will be submitted to the Human Research Ethics Committee (HREC) at the University of Witwatersrand and the Population Council Institutional Review Board. Protection of human subjects will comply with USAID policy. In addition, permission will be sought from communities where the research is conducted.

The confidentiality of all participants enrolled in this research will be protected to the fullest extent possible. All staff involved in this study will receive appropriate training on research ethics, emphasizing the importance of informed consent and confidentiality. Interviewers will administer questionnaires in privacy. Completed questionnaires will be uploaded from the PDA each day to the central server housed at MatCH Research Unit’s offices. Electronic data will be encrypted and stored in password protected files.

All study records will be kept in a locked file cabinet accessible only to authorized members of the study team. All computer entry and networking programs will identify participants only by coded identification numbers. Interviewers will record the participant’s contact information directly into an encrypted and password protected file, where it will be linked to a unique study identification number. The list linking participant identification numbers to other identifying information will be stored separately from coded study forms and source records, in a locked file in a room with limited access in MatCH Research Unit’s office.

Data will be reported aggregately and participants will not be identified by name in any report or publication resulting from data collected in this study. Council staff will conduct monitoring visits to observe fieldwork and ensure interviewers’ adherence with confidentiality procedures. Match Research Unit will store study records until two years after publication of study findings, after which time records will be destroyed. If there is no publication, recordings will be erased no later than six years after the study has ended.

***Compensation***

Both female and male participants will be compensated for their time and for any travel costs related to participating in this research study. This is based on current participant compensation rates used by MatCH Research Unit in similar studies. Women and men who enroll in the cohort study and complete the survey will be paid R100 (~$14.00) for each round of data collection. If there is substantial loss to follow-up, we will consider increasing the compensation further for later rounds (to approximately R150 (~$21.00).

## Routine program monitoring data

A few additional sources of information will be collected and analyzed, to supplement the primary data collection. This will include a secondary data analysis of (1) monitoring data collected by the intervention partner CCI, and (2) clinical data tracked by local systems (or DHIS). No identifier records will be collected, recorded, or stored for this data.

- *CCI monitoring data:*
  We will supplement self-reported data about program exposure with CCI monitoring data that tracks community (cluster)-level program exposure. Such information would largely be aggregated at the community level and will, for example, include information about the number of community leaders engaged by the program, whether the community has developed an activity plan, the percent of individuals from the community engaging in different components of the CR program, and the number of outreach events conducted. Attendance data at CR organized meetings will also be reviewed. The monitoring information will be triangulated with survey responses, whenever possible.
- *DHIS data:*
  Data will be collected from health monitoring information systems (DHIS), which include the number of clients served for indicators including HTC, condom distribution, ART and STI services. Where possible, the data will be analyzed from facilities most proximal to the study locations, both prior to and during the program period. DHIS data is collated per month and while relating clinic data to clusters or individuals will not be possible, we will explore historic trends in service uptake and seek to connect them with program implementation. Data will be analyzed for changes in from baseline to end of the study follow-up and between exposed and control communities. Information on the prevalence of violence in the communities will be obtained, where possible, from the South African Policy Service. Such indicators would include general rates of violence as well as gender-based violence.

## Implementation Science (IS)

We propose to address critical IS research questions focused on gaining a deeper understanding of the process, experiences, challenges, and benefits of adapting and scaling up the Stepping Stones intervention among adults in informal settlements of KwaZulu-Natal. Results would be used to inform development, adaptation, and scale-up of similar interventions.

Primarily, the IS will focus on lessons learned about scaling this intervention (with nongovernmental organizations which are less familiar with implementing this intervention). This includes lessons learned from scaling up the intervention from a smaller controlled clinical trial to a much larger program with new implementers in a new setting. We would address the following primary question:

How do implementers and participants respond to the length and intensity of the scaled-down version of the Stepping Stones manual used in this program?

Other questions in this assessment include:

- What training is received by the facilitators, and are the facilitators able to effectively deliver the content?
- How have program implementers updated the SS intervention to incorporate current ARV and PrEP guidelines, and how do participants react to this information?
- What are the major program challenges and successes, from the perspective of both program implementers and participants?

To answer these questions we propose to use several sources of data:

- IDIs and FGDs with purposively chosen men and women from the study population
- IDIs and FGDs with program staff
- Observation of selected Stepping Stones sessions
- CCI monitoring data and reports

### IDIs and FGDs with cohort participants

#### a. Study population

The research team will conduct a series of qualitative interviews with selected study participants. Two rounds of qualitative interviews are planned. In each round, the following participants will be recruited.

**IDIs**

- 15 women per round
- 15 men per round

**FGDs**

- 1 FGD with 10 women
- 1 FGD with 10 men
- 1 FGD with approximately 5 women and 5 men

***Inclusion criteria***

- Female aged 18–25 or male aged 18–34
- Willing and able to provide fully informed consent
- Lives in community selected for this study and has participated in at least one survey after intervention exposure
- Has participated in the CR Stepping Stones intervention

***Exclusion criteria***

- Unwillingness to have discussion recorded

#### b. Study procedures

The research team will conduct IDIs and FGDs among subset of participants in the household surveys. Research assistants trained in qualitative interviewing methods will conduct these interviews at two points in time. The first set of qualitative interviews will take place after the intervention is rolled out in the first cluster—approximately June and July 2017. The second set of qualitative interviews will take place in February and March 2019 during endline data collection.

Survey participants will be selected for interview based on their responses to questions assessing exposure to the CR intervention. Once identified, the team will contact selected IDI and FGD candidates and invite them to participate in an IDI or FGD that will last 60–90 minutes. The interviewers will thoroughly explain the study, obtain informed consent. To facilitate IDIs and stimulate conversation in FGDs, interviewers will use semi-structured IDI (Annex G) and FGD (Annex H) guides that ask questions about the participants’ experiences with the program.

IDIs will take place in a private location within (or near) the participant’s home, where their responses cannot be overheard. If they do not wish to be interviewed at home, participants may choose to be interviewed at an alternative community venue or MatCH Research offices. FGDs will be conducted in a convenient community venue that permits space for a group discussion that will not be overheard by others.

#### c. Risks and benefits

*Risks:*

There are minimal risks associated with participating in these interviews. As a condition of participation, the interviewer will ask permission from each participant to digitally record the discussions. The interviewers will assure the participants that all measures are taken to ensure the security of these recordings. Interviewers will also tell participants that their viewpoints will not be shared with others, and that they will not be subjected to any repercussions for voicing their opinions on the project. However, while FGD participants will be asked to keep the discussion confidential, there is a risk that FGD participants might breach participant confidentiality agreements after the discussion.

*Benefits:*

No immediate tangible benefit is likely to accrue to the subjects through their participation and this will be made clear when obtaining informed consent. However the interviewers will describe the potential benefits of conducting this study, for health care systems and clients thereof, so that prospective participants are fully aware of how the data gathered will be used to provide programmatic recommendations and help others in their community.

#### d. Confidentiality

#### The confidentiality of all participants enrolled in this research will be protected to the fullest extent possible. The importance of FGD confidentiality will be discussed with participants before the discussion begins. Participants will be identified only by their unique study ID number in the recording of the interview / discussion; the recorded conversation and transcripts will contain no information that could link the recording to any individual participant. Digital recordings will be stored in password protected files on MatCH Research Unit servers. Data will be reported aggregately and participants will not be identified by name in any report or publication resulting from data collected in this study. Council staff will conduct monitoring visits to observe fieldwork and ensure interviewers’ adherence with confidentiality procedures.

#### e. Compensation

Both female and male participants will be compensated for their time related to participating in this research study. This is based on current participant compensation rates used by MatCH Research in similar studies. Women and men will be paid R100 (~$14.00) per interview for participating in a FGD or IDI.

#### f. Informed consent process

Although participants were informed of the possibility that they might be asked to participate in an IDI or FGD, the research team will obtain separate informed consent for this activity. Immediately before the IDI or FGD, the interviewer will explain the study goals, risks, and benefits. If the participant is interested, the interviewer will provide her/him with the appropriate IDI (Annex B) or FGD (Annex C) informed consent document, reading the IDI informed consent aloud if necessary.

The interviewer will answer all questions posed by the participant about the study, emphasizing that the study is voluntary, confidential, and the participant can end the interview at any time. The interviewer will also emphasize that all information collected will be kept confidential, and that no identifying information will be linked to their responses. Furthermore, neither the participant’s decision to participate, nor their responses to the questions asked, will in any way affect their standing with the CR program. Participants will be asked to provide written consent and the interviewer will countersign the informed consent form, offering the participant will be offered a copy of the signed informed consent form.

### IDIs and FGDs with Community Responses staff

#### a. Study population

Data collection will take place at two separate time periods, each including two FGDs with program staff and facilitators, plus five IDIs with key representatives from program management. These discussions and interviews will be conducted after the first round of implementation (i.e., at the end of the first stepped wedge/approximately ten months after the initial baseline), and at the end of the full implementation period/at end line.

The research team will conduct a series of qualitative interviews with staff who work with the Stepping Stones component of the CR program. Two rounds of qualitative interviews are planned. In each round, the following participants will be recruited.

**IDIs:**

- 5 CR program managers, who oversee/work with the Stepping Stones component

**FGDs**

- 2 FGDs with 10 Stepping Stones facilitators and program staff

***Inclusion criteria***

- Female or male, over age 18
- Willing and able to provide fully informed consent
- Works with the Stepping Stones component as part of the CR program

***Exclusion criteria***

- Unwillingness to have discussion recorded

b. Study procedures

The research team will conduct IDIs and FGDs among staff of the CR program. Research assistants trained in qualitative interviewing methods will conduct these interviews at two points in time. The first set of qualitative interviews will take place after the intervention is rolled out in the first cluster—approximately June and July 2017—and as part of the second round of data collection in the first cluster. This will permit early reactions to the program, in addition to the reactions/observations collected at endline. The second set of qualitative interviews will take place in February and March 2019 during endline data collection.

The research team will identify potential participants who meet eligibility criteria and invite them to participate in an IDI or FGD, where they will be asked to discuss their work on the project. Interviews will be digitally recorded, and trained interviewers will use semi-structured IDI (Annex I) or FGD (Annex J) guides to facilitate discussion. These interview guides contain questions about participant experiences with the program. Interviews will last 60–90 minutes.

To maximize the confidentiality of participant responses, interviewers will conduct interviews in a quiet, private space selected with the input of the participant. Conversations will be recorded digitally. This study activity is scheduled to occur concurrently with endline survey data collection, and will last approximately 2–3 weeks.

c. Risks and benefits

*Risks:*

There are minimal risks associated with participating in these interviews. As a condition of participation, the interviewer will ask permission from each participant to digitally record the discussions. The interviewers will assure the participants that all measures are taken to ensure the security of these recordings. Interviewers will also tell participants that their viewpoints will not be shared with others, and that they will not be subjected to any repercussions for voicing their opinions on the project. However, while FGD participants will be asked to keep the discussion confidential, there is a risk that FGD participants might breach participant confidentiality agreements after the discussion.

*Benefits:*

No immediate tangible benefit is likely to accrue to the subjects through their participation and this will be made clear when obtaining informed consent. However the interviewers will describe the potential benefits of conducting this study, for health care systems and clients thereof, so that prospective participants are fully aware of how the data gathered will be used to provide programmatic recommendations and help others in their community.

d. Confidentiality

#### The confidentiality of all participants enrolled in this research will be protected to the fullest extent possible. The importance of FGD confidentiality will be discussed with participants before the discussion begins. Participants will be identified only by their unique study ID number in the recording of the interview; the recorded conversation and transcripts will contain no information that could link the recording to any individual participant. Digital recordings will be stored in password protected files on MatCH Research Unit servers. Data will be reported in aggregate; participants will not be reported by name in any report or publication resulting from data collected in this study. Council staff will conduct monitoring visits to observe fieldwork and ensure interviewers’ adherence with confidentiality procedures.

e. Compensation

Program staff will be compensated for their time related to participating in this research study. This is based on current participant compensation rates used by MatCH Research in similar studies. Program staff will be paid R100 (~$14.00) per interview.

f. Informed consent process

Although participants were informed of the possibility that they might be asked to participate in an IDI or FGD, the research team will obtain separate informed consent for this activity. Immediately before the IDI or FGD, the interviewer will explain the study goals, risks, and benefits. If the participant is interested, the interviewer will provide her/him with the appropriate IDI (Annex D) or FGD (Annex E) informed consent document, reading the IDI informed consent aloud if necessary.

The interviewer will answer all questions posed by the participant about the study, emphasizing that the study is voluntary, confidential, and the participant can end the interview at any time. The interviewer will also emphasize that all information collected will be kept confidential, and that no identifying information will be linked to their responses. Furthermore, neither the participant’s decision to participate, nor their responses to the questions asked, will in any way affect their standing with the CR program. Participants will be asked to provide written consent and the interviewer will countersign the informed consent form, offering the participant will be offered a copy of the signed informed consent form.

### Session Observations

#### Study procedures

We will also conduct 5–10 observations of Community Responses sessions to assess program quality and answer questions posed in the IS section. During an observation, a data collector will attend a scheduled Community Responses session, and take unstructured notes addressing the key IS questions, including those related to program quality, fidelity, and participant response. The observer will not participate in the session, nor will s/he initiate communication with attendees unless necessary; s/he will simply observe and record.

#### Risks and benefits to participants

Risks:

As the observer will neither participate in the session, or collect information about individual attendees, this activity poses minimal risk.

Benefits:

No immediate tangible benefit is likely to accrue to the subjects through their participation and this will be made clear when obtaining informed consent. However the interviewers will describe the potential benefits of conducting this study, for health care systems and clients thereof, so that prospective participants are fully aware of how the data gathered will be used to provide programmatic recommendations and help others in their community.

#### Confidentiality

The observer will not record information about specific attendees.

#### Compensation

This activity does not pose undue inconvenience to session attendees. Thus, compensation is not provided.

#### Informed consent process

Since the observer is completing an unstructured observation, and is unlikely to directly interact with participants, informed consent is unnecessary for this activity. However, as session attendees may be curious about the observer, the facilitator will describe the purpose of the observations to the group before starting the session.

# Qualitative Data Analysis

Qualitative data will be guided by the key Implementation Science questions for this study, and will focus on lessons learned about the implementation of Stepping Stones in this setting. The IDIs, FGDs, and unstructured observations will be reviewed, and themes relating to the updating, intensity, length, and content of the Stepping Stones intervention will be elicited and grouped so that they may assist future programmers/implementers of the program. Particularly insightful stories or quotes will also be extracted from the data sets, to provide more context for the themes produced, and to share important examples.

# Data Management

As stated previously, participants contact information will be collected; however strict measures will be taken to conceal participants’ identities. All questionnaires and digital recordings will be identified only by unique study identification codes; no participant names will be recorded on these forms. All completed data collection instruments will be kept in locked storage cabinets or rooms at all times. Digital recordings will be stored on password-protected computers. The list linking participant identification numbers to other identifying information will be stored separately from coded study forms and source records, in a locked file in a room with limited access in MatCH Research Unit’s office. Participants will not be reported by name in any report or publication resulting from data collected in this study.

The study coordinator will collect all completed data collection instruments each day and deposit them into lockboxes, to which only the data collection team leader and primary in-country research manager will have keys. Hard copies of study documents will be stored securely in locked cabinets for no longer than five years after the study has ended.

Data collectors will use electronic data capture systems such as tablet computers or PDAs to complete study surveys. Completed surveys will be converted into to comma-separated value files for use with statistical analysis software. Electronic data will be stored in files on a secure server in study-specific files, accessible only on password-protected computers. Access to data will be limited to the research team. No identifying details will be included in any final output, and all presented data will be aggregated at the health facility level or higher to ensure that no responses can be traced back to an individual participant.

# Work Plan

## Research utilization strategy

The key audiences for this study are governmental decision-makers, HIV service and program providers, donors, and the communities themselves. We will promote research utilization through active outreach at the start of the study, and periodically throughout. We will plan to enlist an advisory group of relevant stakeholders (e.g., representatives from the South African government, representatives from the HEARD study), conduct a data interpretation workshop to discuss and interpret results, and disseminate final results locally and internationally. We will produce both a full technical report and two manuscripts for publication. Information generated from the study will be shared with the research communities through well-established relationships with Wits University (where our proposed research partner is housed), and other academic institutions in South Africa and internationally. We also will disseminate findings to key stakeholders in KwaZulu-Natal Province, including the Department of Health, as well as nationally. We expect that these results will be generalizable to other settings, in particular similar country settings in Africa. As part of the larger SOAR portfolio, results will also feed into regional and interregional dissemination efforts. We also anticipate that the rigor of this study’s design will evoke substantial interest in the findings among the global community.

## Capacity strengthening activities

The proposed local research partner in this effort, Maternal, Adolescent and Child Health Research (MRU) of the Department of Obstetrics and Gynaecology, University of the Witwatersrand (Wits University), is a quality institution, with a number of well-trained researchers on staff. There are, however, ample opportunities to support senior MRU researchers in training and expanding the skill base of junior researchers at MRU. By working closely with the local team, we will enhance capacity in program monitoring and rigorous program evaluation. We will also strive to include South African students and young investigators in the study to ensure that they learn from this opportunity.

## Personnel and implementing organizations

The Population Council will lead conceptualization, analysis, and write up of the CR program evaluation. Stephanie Psaki (co-PI) will provide expertise in survey design, data analysis, and reporting, as well as the design and evaluation of programs focused on gender and sexual health; Julie Pulerwitz (co-PI) will provide expertise on implementation science around community-based programs, gender norms measurement and analysis, and technical assistance on interventions focused on gender dynamics; and Paul C. Hewett (co-PI) will provide expertise on the evaluation design, sampling, data collection, and data analysis, as well as technical expertise in HIV prevention interventions.

The Population Council has extensive experience evaluating HIV and AIDS programs, including those that address sexual risk behaviors, and promote VMMC/HCT (e.g., Horizons Program—1997–2007; Project SOAR—2014–2019). The Council also has conducted extensive research on structural barriers to HIV prevention, including gender-based power differentials, and GBV (e.g., Pulerwitz et al. 2015). Further, Project SOAR is currently working in South Africa with partners Wits University, University of North Carolina, Chapel Hill (UNC), University of California, San Francisco (UCSF), and Sonke Gender Justice to assess the effects of shifting certain common gender norms on HIV service utilization; the primary outcome is HIV testing.

MRU aims to answer priority questions that will translate into improving sexual and reproductive health outcomes through expanding access to appropriate and acceptable contraceptive, HIV prevention, and related health technologies and services. Using a range of methodologies, MRU conducts behavioural, operations, and clinical research, actively partnering and involving communities and local structures. Collaborators at MRU will contribute to the conceptualization, design, and analysis of evaluation results, and will lead field data collection. Mags Beksinska (co-PI) will assist with the management of study implementation and oversight of the scientific integrity of the evaluation. She will develop study protocols and associated data collection instruments, and will provide oversight of cohort data collection, and ethical oversight for the study.

All lead researchers/institutions will contribute to final products and publications.

## Products

The SOAR CR evaluation will produce:

- Technical report
- At least two manuscripts for a scientific, peer-reviewed journal

**Timeline**

|  | **2015** | **2016** | | | | **2017** | | | | **2018** | | | | **2019** | | |
| --- | --- | --- | --- | --- | --- | --- | --- | --- | --- | --- | --- | --- | --- | --- | --- | --- |
|  | **Q4** | **Q1** | **Q2** | **Q3** | **Q4** | **Q1** | **Q2** | **Q3** | **Q4** | **Q1** | **Q2** | **Q3** | **Q4** | **Q1** | **Q2** | **Q3** |
| Proposal review and approval |  |  |  |  |  |  |  |  |  |  |  |  |  |  |  |  |
| Protocol development, finalization, and submission to local and Council IRBs (May 2016) |  |  |  |  |  |  |  |  |  |  |  |  |  |  |  |  |
| Anticipated approval from local and Council IRBs |  |  |  |  |  |  |  |  |  |  |  |  |  |  |  |  |
| Intervention start (staged rollout) in evaluation areas |  |  |  |  |  |  |  |  |  |  |  |  |  |  |  |  |
| Baseline/comparison data collection in evaluation areas |  |  |  |  |  |  |  |  |  |  |  |  |  |  |  |  |
| IS/qualitative interviews |  |  |  |  |  |  |  |  |  |  |  |  |  |  |  |  |
| Data cleaning, analysis, and write up |  |  |  |  |  |  |  |  |  |  |  |  |  |  |  |  |
| Dissemination |  |  |  |  |  |  |  |  |  |  |  |  |  |  |  |  |

Note: Timeline only refers to implementation in KwaZulu-Natal Province. Implementation of the CR program in remaining provinces will depend on CCI timeline, and will not be affected by the timing of IRB approval and data collection for this evaluation.

# References

Auvert B, Taljaard D, Lagarde E, Sobngwi-Tambekou J, Sitta R, et al. 2005. Randomized, controlled intervention trial of male circumcision for reduction of HIV infection risk: The ANRS 1265 trial. *PLoS Med.* 2(11): e298. doi: 10.1371/journal.pmed.0020298.

Baio G, Copas A, Ambler G, Hargreaves J, Beard E and R Z Omar. 2015. Sample size calculation for a stepped-wedge trial. *Trials*. 16(354). doi:10.1186/s13063-015-0840-9.

Bailey RC, Moses S, Parker CB, Agot K, Maclean I, Krieger JN et al 2007. Male circumcision for HIV prevention in young men in Kisumu, Kenya: a randomised controlled trial. Lancet. 369(9562):643-56.

Brown C & Lilford RJ. 2006. The stepped-wedge trial design: a systematic review. *BMC Medical Research Methodology*. 6:54. doi: 10.1186/1471-2288-6-54.

Dworkin S, Treves-Kagan S, Lippman S. 2013. Gender-transformative interventions to reduce HIV risks and violence with heterosexually-active men: a review of the global evidence. *AIDS and Behavior*. 17(9):2845–63.

Gomez AM, Speizer IS, Moracco KE. 2011. Linkages between gender equity and intimate partner violence among urban Brazilian youth. *Journal of Adolescent Health*. 49, pp 393-399.

Gray RH, Kigozi G, Serwadda D, Makumbi F, Watya S, Nalugoda F et al 2007. Male circumcision for HIV prevention in men in Rakai, Uganda: a randomised trial. *Lancet*. 369(9562):657-66.

Hemming, K., T.P. Haines, P.J. Chilton, A.J. Girling, R.J. Lilford 2015. “The stepped-wedge cluster randomized trial: rationale, designs, analysis and reporting.” BMJ 2015; 350: h391.

Hemming, Karla and Alan Girling. 2014. A menu-driven facility for power and detectable-difference calculations in stepped-wedge cluster-randomized trials. *The Stata Journal*. 14(2): 363-380.

Hughes JP, Goldenberg RL, Wilfert CM. et al. 2003. “Design of the HIV prevention trials network (HPTN) protocol 054: a cluster randomized crossover trial to evaluate combined access to nevirapine in developing countries,” *Technical Report 195*. Seattle: University of Washington, Department of Biostatistics.

Hussey MA & Hughes JP. 2007. Design and analysis of stepped-wedge cluster randomized trials. 28(2):182-91.

Jewkes R, Nduna M, Jama N, Puren A, Duvvury N. 2008. “Impact of stepping stones on incidence of HIV and HSV-2 and sexual behaviour in rural South Africa: cluster randomised controlled trial.” *BMJ* 337:a506

Karim AM, Magnani RJ, Morgan GT, Bond KC. 2003. Reproductive health risk and protective factors among unmarried youth in Ghana. *International Family Planning Perspectives* 29(1):14-24.

[Lippman SA](http://www.ncbi.nlm.nih.gov/pubmed/?term=Lippman%20SA%5BAuthor%5D&cauthor=true&cauthor_uid=24147121), [Lippman SA](http://www.ncbi.nlm.nih.gov/pubmed/?term=Lippman%20SA%5BAuthor%5D&cauthor=true&cauthor_uid=24147121), [Maman S](http://www.ncbi.nlm.nih.gov/pubmed/?term=Maman%20S%5BAuthor%5D&cauthor=true&cauthor_uid=24147121), [MacPhail C](http://www.ncbi.nlm.nih.gov/pubmed/?term=MacPhail%20C%5BAuthor%5D&cauthor=true&cauthor_uid=24147121), [Twine R](http://www.ncbi.nlm.nih.gov/pubmed/?term=Twine%20R%5BAuthor%5D&cauthor=true&cauthor_uid=24147121), [Peacock D](http://www.ncbi.nlm.nih.gov/pubmed/?term=Peacock%20D%5BAuthor%5D&cauthor=true&cauthor_uid=24147121), [Kahn K](http://www.ncbi.nlm.nih.gov/pubmed/?term=Kahn%20K%5BAuthor%5D&cauthor=true&cauthor_uid=24147121), [Pettifor A](http://www.ncbi.nlm.nih.gov/pubmed/?term=Pettifor%20A%5BAuthor%5D&cauthor=true&cauthor_uid=24147121). 2013 “Conceptualizing community mobilization for HIV prevention: implications for HIV prevention programming in the African context,” [*PLoS One*.](http://www.ncbi.nlm.nih.gov/pubmed/?otool=uncchlib&term=Lippman%20Pettifor) Oct 11;8(10):e78208. doi: 10.1371/journal.pone.0078208.

Mantell J, Cornish F and J Russell. 2014. Evaluating social outcomes of HIV/AIDS interventions: a critical assessment of contemporary indicator frameworks. *Journal of the International AIDS Society*. 17:19073. <http://dx.doi.org/10.7448/IAS.17.1.19073>

Pulerwitz, J, Barker, G. 2008. Measuring attitudes toward gender norms among young men in Brazil: Development and psychometric evaluation of the GEM Scale. *Men and Masculinities*. 10: 322-338.

Pulerwitz, J, Michaelis, A, Verma, R, Weiss, E. 2010. Addressing gender dynamics and engaging men in HIV programs: Lessons learned from Horizons research. *Public Health Reports*. 125: 282–292.

Pulerwitz, J, Barker, G, Verma, R. 2012. Changing gender norms for HIV and violence risk reduction, in *The Handbook of Global Health Communication* (eds R. Obregon and S. Waisbord), Wiley-Blackwell, Oxford, UK.

Pulerwitz, J, Hughes, L, Mehta, M, Kidanu, A, Verani, F, Tewolde, S. 2015. Changing gender norms and reducing intimate partner violence: results from a quasi-experimental intervention study with young men in Ethiopia. *American Journal of Public Health* 105(1): 132–137.

South African National AIDS Council (SANAC). 2011. South Africa’s National Strategic Plan (NSP) on HIV and AIDS, STIs and TB for 2012-2016. Pretoria: SANAC.

Sgaier SK, Reed JB, Thomas A & Njeuhmeli E. May 2014. Achieving the HIV prevention impact of voluntary medical male circumcision: lessons and challenges for managing programs. *PLoS Medicine*. 11(5).

Shannon K, Leiter K, Phaladze, et al. 2012. Gender inequity norms are associated with increased male-perpetrated rape and sexual risks for HIV infection in Botswana and Swaziland. *PLoS ONE* 7(1): e28739. doi:10.1371/journal.pone.0028739

Shisana, O, Rehle, T, Simbayi LC, et al. 2014. South African National HIV Prevalence, Incidence and Behaviour Survey, 2012.

Stephenson R, Bartel D, Rubardt M. 2012. Constructs of power and equity and their association with contraceptive use among men and women in rural Ethiopia and Kenya. *Global Public Health*, Vol 7, No. 6, pp 618-634.

Tang CS, Wong C, Lee AM. 2001. Gender-related psychosocial and cultural factors associated with condom use among Chinese married women. *AIDS Education and Prevention* 13(4):329-342

1. Although this is the focal population for the intervention, CCI has indicated that the program will not be limited to this age group. [↑](#footnote-ref-2)
2. PEPFAR Level 1 Indicator definition for PP_PREV: Percentage of individuals from priority populations who completed a standardized HIV prevention intervention, including the specified minimum components, during the reporting period. [↑](#footnote-ref-3)
3. ZAZI is a local Nguni word meaning “know yourself.” ZAZI is a USAID supported program to promote self-confidence and efficacy among women to prevent unwanted pregnancy, prevent HIV and improve maternal and child health through safe pregnancies. [↑](#footnote-ref-4)
4. Brothers for Life is a USAID support program to promote HIV prevention through promotion of VMMC, HTC, safer sexual behavior and a reduction in gender based violence. The program also engages in activities to promote and improve men’s sexual and reproductive health. [↑](#footnote-ref-5)
5. PEPFAR Level 1 indicator definition for GEND_NORM: Number of people completing an intervention pertaining to gender norms that meets minimum criteria. [↑](#footnote-ref-6)
6. SKILLz is a lifeskills and sexual reproductive health curriculum that provides young boys and girls 12–18 knowledge and decision making around HIV prevention, discrimination, stigma, HIV testing and access to treatment. [↑](#footnote-ref-7)
7. Stepping Stones is a program in which community members are engaged in interactive sessions covering topics on gender, HIV, communication and relationships skill building. In addition, Stepping Stones addresses issues of gender norms, gender-based violence and intimate partner violence. [↑](#footnote-ref-8)
8. Center for Communication Impact, “USAID Community Responses: Overview of the CR Programme.” Sub-partner Inception Meeting. 12 November 2015, South Africa. [↑](#footnote-ref-9)
9. Conducting “as treated” analyses on an individual-level will require use of appropriate study design and statistical approaches for handling self-selection into program exposure groups, e.g., random promotion or instrumental variable analysis. [↑](#footnote-ref-10)
10. Statistical power in stepped-wedge design trials depends on the number of clusters, the number of randomization times, and the number of clusters randomized at each time point; increasing the overall number of measurement times (i.e., randomization times) increases power. Power is also affected by amount of time needed for full effect of program; that is, if the full effects are not realized in the first time period in which it is implemented, power will be further reduced (Hussey & Hughes 2007). [↑](#footnote-ref-11)
11. Wards are geopolitical subdivisions of municipalities. Although defined largely for electoral purposes, wards are a geographic level of demarcation use by the South Africa National Census. [↑](#footnote-ref-12)
12. Google Earth provides high resolution satellite images of at least 15 meters per pixel of resolution, providing relative clear demarcations of structures and landmarks. Most images of the informal settlements and environs in South Africa are from 2016 and, hence, up-to-date. [↑](#footnote-ref-13)
